# On Multimatrix Models Motivated by Random Noncommutative Geometry II: A Yang-Mills-Higgs Matrix Model

**DOI:** 10.1007/s00023-021-01138-w

**Published:** 2022-04-23

**Authors:** Carlos I. Perez-Sanchez

**Affiliations:** 1 Faculty of Physics, University of Warsaw, ul. Pasteura 5, 02-093 Warsaw, Poland; 2 Institute for Theoretical Physics, University of Heidelberg, Philosophenweg 19, 69120 Heidelberg, Germany

## Abstract

We continue the study of fuzzy geometries inside Connes’ spectral formalism and their relation to multimatrix models. In this companion paper to Pérez-Sánchez (Ann Henri Poincaré 22:3095–3148, 2021, arXiv:2007.10914), we propose a gauge theory setting based on noncommutative geometry, which—just as the traditional formulation in terms of almost-commutative manifolds—has the ability to also accommodate a Higgs field. However, in contrast to ‘almost-commutative manifolds’, the present framework, which we call gauge matrix spectral triples, employs only finite-dimensional algebras. In a path-integral quantization approach to the Spectral Action, this allows to state Yang–Mills–Higgs theory (on four-dimensional Euclidean fuzzy space) as an explicit random multimatrix model obtained here, whose matrix fields exactly mirror those of the Yang–Mills–Higgs theory on a smooth manifold.

## Introduction

The approximation of smooth manifolds by *finite* *geometries* (or geometries described by finite-dimensional algebras) has been treated in noncommutative geometry (NCG) some time ago [36] and often experiences a regain of interest; in [20, 25], for instance, these arise from truncations of space to a finite resolution. In an ideologically similar vein but from a technically different viewpoint, this paper addresses gauge theories derived from the Spectral Formalism of NCG, using exclusively finite-dimensional algebras, also for the description of the space(time). This allows one to make precise sense of path integrals over noncommutative geometries. Although this formulation is valid at the moment only for a small class of geometries, the present method might shed light on the general problem of quantization of NCG, already tackled using von Neumann’s information theoretic entropy in [15, 24], by fermionic and bosonic-fermionic second quantization, respectively.

Traditionally, in the NCG parlance, the term ‘finite geometry’ is employed for an extension of the *spacetime* or *base manifold* (a spin geometry or equivalently [18, 48] a *commutative spectral triple*) by what is known in physics as ‘inner space’ and boils down to a choice of a Lie group (or Lie algebra) in the principal bundle approach to gauge theory. In contrast, in the NCG framework via the Spectral Action [13], this inner space—called *finite geometry* and denoted by *F*—is determined by a choice of certain finite-dimensional algebra whose purpose is to encode particle interactions; by doing so, NCG automatically rewards us with the Higgs field. Of course, the exploration of the right structure of the inner space *F* is also approached using other structures, e.g., non-associative algebras [7, 9, 27, 54] for either the Standard Model or unified theories, but in this paper we restrict ourselves to (associative) NCG-structures.

**Fig. 1:**
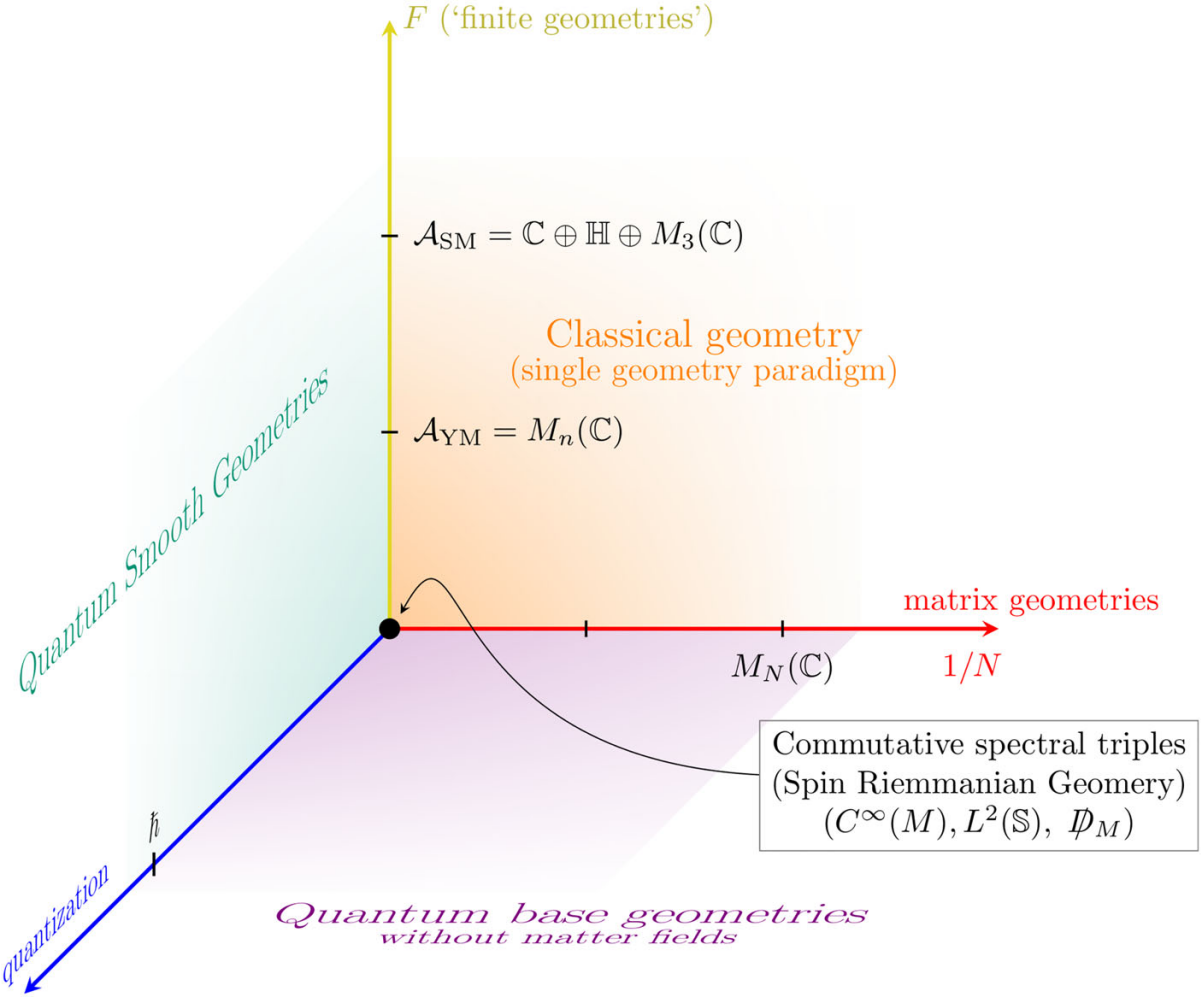
Three axis representing independent theories (all inside NCG), starting from spin Riemannian geometry at the origin. Abbreviations and terminology: YM = Yang–Mills; SM = Standard Model

Still in the traditional approach via *almost-commutative geometries* $M\times F$ [14, 52, 57], the finite geometry *F* plays the role of discrete extra dimensions or ‘points with structure’ extending the (commutative) geometry *M*, hence the name. What is different in this paper is the replacement of smooth spin geometries *M* by a model of spacetime based on finite-dimensional geometries (‘finite spectral triples’) known as *matrix geometry* or *fuzzy geometry* [3]. Already at the level of the classical action, these geometries have some disposition to the quantum theory, as it is known from well-studied ‘fuzzy spaces’ [23, 37, 49–51, 53], which are not always based on Connes’ formalism.^1^ This article lies in the intersection and treats ‘fuzzy spaces’ inside the Spectral Formalism.

At this point, it is pertinent to clarify the different roles of the sundry finite-dimensional algebras that will appear. Figure 1 might be useful to illustrate why matrix algebras that differ only in size are given different physical nature. In this cube, pictorially similar to Okun’s ‘cube for natural units’ [28, 40], classical Riemannian geometry sits at the origin (0, 0, 0). Several NCG-based theories of physical interest may have, nevertheless, the three more general coordinates $(\hbar ,1/N,F)$ described now:

$$\bullet $$
The *F*-direction in Fig. 1 describes (bosonic) matter fields. Mathematically the possible values for *F* correspond to a ‘finite geometry.’ These were classified by Paschke and Sitarz [43] and diagrammatically by Krajewski [35]. Particle physics models based on NCG and the Connes–Chamseddine spectral action [2, 6, 14, 19, 21, 25] ‘sit along the *F*-axis’. From those spectral triples *F*, only their algebra appears in Fig. 1.
$$\bullet $$
A finite second coordinate, $1/N>0$, means that the smooth base manifold that encodes space(time) has been replaced by a ‘matrix geometry,’ which in the setting [3] is a spectral triple based on an algebra of matrices of size *N* (and albeit finite-dimensional, escaping Krajewski’s classification).
$$\bullet $$
The remaining coordinate denotes quantization when $\hbar \ne 0$. In the path integral formalism, the partition function is a weighted integral $Z=\int _{} \mathrm {d}\xi \, \mathrm {e}^{\mathrm {i}S(\xi )/\hbar }$ over the space of certain class of geometries $\xi$, the aim being the quantization of space itself, having quantum gravity as motivation. Here *S* is the classical action.

Accordingly, the planes orthogonal to the axis just described are:

$$\bullet $$
*The plane* $(\hbar ,1/N,0)$ *of base geometries*. On the marked plane orthogonal to *F* lie ‘spacetimes’ or^2^ ‘base manifolds’ and, when these are not flat, they can model gravity degrees of freedom. If $F=0$, no gauge fields live on such space.
$$\bullet $$
*The plane* $(\hbar , 0, F)=\lim _{N\rightarrow \infty } (\hbar ,1/N,F)$. On the plane orthogonal to the ‘matrix geometry’ axis, one has the quantum, smooth geometries (meaning, their algebra is or contains a $C^\infty (M)$ as factor). The long-term aim is to get to the ‘quantum smooth geometry plane’ as matrix algebras become large-dimensional, which is something that, at least for the sphere, is based on sound statements [44–47] in terms of Gromov–Hausdorff convergence. Additional to such large-*N*, one might require to adjust the couplings to criticality [8, 29, 34]. This can also be addressed using doubly scaling limits together with the Functional Renormalization Group to find candidates for phase transition; for models still without matter, see [42].
$$\bullet $$
*The plane* $(0,1/N,F)=\lim _{\hbar \rightarrow 0} (\hbar , 1/N,F)$ *of classical geometries*. By ‘classical geometry’ we mean a single geometrical object (e.g., a Lorentzian or Riemannian manifold, a $\mathrm {SU}(n)$-principal bundle with connection, etc.), which can be determined by, say, the least-action principle (Einstein Equations, $\mathrm {SU}(n)$-Yang–Mills Equations, etc.). This is in contrast to the quantization of space, which implies a multi-geometry paradigm, at least in the path integral approach.

**Fig. 2:**
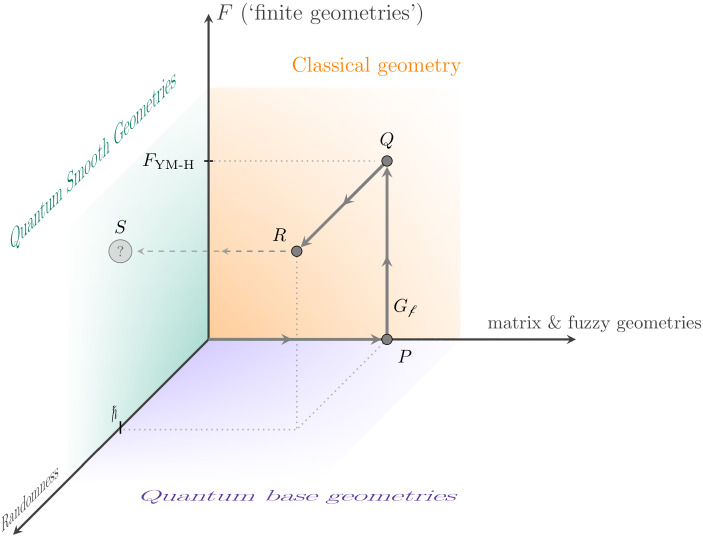
Depicting the organization of this article, following the path *PQR*. Here, $F_{{\textsc {ym}}\text {-}{\textsc {h}}}={(M_n({\mathbb {C}}), M_n({\mathbb {C}}),D_F)}$ corresponds to the spectral triple for the Yang–Mills–Higgs theory and ${G_\mathrm {f}}$ is a fuzzy four-dimensional geometry. As outlook (dashed), to reach a smooth geometry at the point *S* one needs a sensible limit (e.g., large-*N* and possibly tuning some parameters to criticality) in order to achieve phase transition

The program started here is not as ambitious as to yield physically meaningful results in this very article, but it has the initiative to apply three small steps—one in each of the independent directions away from classical Riemannian geometry—and presents a model in which the three aforementioned features coexist. This paves the way for NCG-models of quantum gravity coupled to the rest of the fundamental interactions (it is convenient to consider the theory as a whole, due to the mutual feedback between matter and gravity sectors in the renormalization group flow; cf. [22] for an asymptotic safety picture). For this purpose, we need the next simplifications, as illustrated in Fig. 2:

$$\bullet $$
Our choice for the finite geometry *F* is based on the algebra ${\mathcal {A}}_F=M_n({\mathbb {C}})$ ($n\ge 2$). This is the first input, aiming at a $\mathrm {SU}(n)$ Yang–Mills theory.
$$\bullet $$
Instead of the function algebra on a manifold, we take a simple matrix algebra $M_N({\mathbb {C}})$. This is an input too. (Also *N* is large and *n* need not be.)
$$\bullet $$
We use random geometries instead of honest quantum geometries; this corresponds with a Wick rotation from $\mathrm {e}^{\mathrm {i}S(\xi )/ \hbar }$, in the partition function, toward the Boltzmann factor $\mathrm {e}^{- S(\xi )/\hbar }$. This setting is often referred to as *random noncommutative geometry* [5, 29].

Random NCG was introduced in [8]. While aiming at numerical simulations for the Dirac operators, Barrett–Glaser stated the low-dimensional geometries as a random matrix model. The Spectral Action of these theories was later systematically computed for general dimensions and signatures in [41]. Also, in the first part of this companion paper, the Functional Renormalization Group to multimatrix models [42] inspired by random noncommutative geometry was addressed for some two-dimensional models obtained in [41]. Solution of the matrix-models corresponding to one-dimensional geometries was addressed in [1], using Topological Recursion [26] (due to the presence of multitraces, in its blobbed [10] version).

The organization of the article is as follows. Next section introduces fuzzy geometries as spectral triples and gives Barrett’s characterization of their Dirac operators in terms of finite matrices. Section 3 interprets these as variables of a ‘matrix spin geometry’ for the (0, 4)-signature. Section 4 introduces the main object of this article, *gauge matrix spectral triples*, for which the spectral action is identified with Yang–Mills theory, if the piece $D_F$ of Dirac operator along the ‘inner space spectral triple’^3^ vanishes, and with Yang–Mills–Higgs theory, if this is non-zero, $D_F\ne 0$ (see Sect. 5). Our cutoff function *f* appearing in the Spectral Action ${{\,\mathrm{Tr}\,}}_{\mathcal {H}}f(D)$ is a polynomial *f* (instead of a bump function^4^). In Sect. 6, we make the parallel of the result with ordinary gauge theory on smooth manifolds. Finally, Sect. 7 gives the conclusion and Sect. 8 the outlook, while also stating the explicit Yang–Mills–Higgs matrix model for further study.

This article is self-contained, but some familiarity with spectral triples helps. Favoring a particle physics viewpoint, we kept the terminology and notation compatible with [57].

## Spectral Triples and Fuzzy Geometries

Let us start with Barrett’s definition of fuzzy geometries that makes them fit into Connes’ spectral formalism.

### Definition 2.1

A *fuzzy geometry* is determined by

$$\bullet $$
a *signature* $(p,q)\in {\mathbb {Z}}_{\ge 0}$, or equivalently, by
  $$\begin{aligned} \eta =\mathrm {diag}(\underbrace{+,\ldots ,+}_{p}, \underbrace{-,\ldots ,-}_q)=\mathrm {diag}(+_p,-_q) \end{aligned}$$
$$\bullet $$
three signs $\epsilon , \epsilon ',\epsilon ''\in \{-1,+1\}$ fixed through *s* by the following table:

**Table:** 

$s\equiv q-p \,\,\mathrm { mod }\, 8$	0	1	2	3	4	5	6	7
$$\epsilon $$	$$+$$	$$+$$	−	−	−	−	$$+ $$	$$+ $$
$\epsilon '$	$+$	−	$+$	$+$	$+$	−	$+$	$+$
$\epsilon ''$	$+$	+	−	+	$+$	+	−	+

$$\bullet $$
a matrix algebra $${{\mathcal {A}}_{\mathrm {f}}=M_N({\mathbb {C}})}$$
$$\bullet $$
a Clifford ${\mathcal {C}}\ell (p,q)$-module *V* or *spinor space*
$$\bullet $$
a *chirality* ${\gamma _{\mathrm {f}}=\gamma \otimes 1_{\mathcal {A}}:{\mathcal {H}}_{\mathrm {f}}\rightarrow {\mathcal {H}}_{\mathrm {f}}}$ for the vector space ${{\mathcal {H}}_{\mathrm {f}}= V\otimes M_N({{\mathbb {C}}})}$ with inner product
  $$\begin{aligned} \langle v\otimes T, w\otimes W \rangle = (v,w) {{\,\mathrm{Tr}\,}}_{N}(T^*W)\, \text {}\, \end{aligned}$$
  for all $T,W\in M_N({\mathbb {C}})$ and $v,w\in V$. To wit $\gamma :V\rightarrow V$ is self-adjoint with respect to the Hermitian form $(v,w)= \sum _a {{\bar{v}}}_a w_a$ on $V \cong {\mathbb {C}}^{k}$ and satisfying $\gamma ^2=1$. This *k* is so chosen as to make *V* irreducible for even *s*. Only the $\pm 1$-eigenspaces of *V* with the grading $\gamma$ are supposed to be irreducible, if *s* is odd
$$\bullet $$
a left-${{\mathcal {A}}_{\mathrm {f}}}$ *representation* on ${{\mathcal {H}}_{\mathrm {f}}}$, $\varrho (a)(v\otimes W) = v\otimes (a W)$, for ${a\in {\mathcal {A}}_{\mathrm {f}}}$ and $W\in M_N({\mathbb {C}})$. The representation $\varrho$ is often implicit
$$\bullet $$
an anti-linear isometry, called *real structure*, ${J_{\mathrm {f}}:=C\otimes *:{\mathcal {H}}_{\mathrm {f}}\rightarrow {\mathcal {H}}_{\mathrm {f}}}$ given in terms of the involution $*$ (in physics represented by $\dagger$) on the matrix algebra and $C:V\rightarrow V$ an anti-linear operator satisfying, for each gamma matrix,
  2.1 $$\begin{aligned} C^2= \epsilon \text{ and } \gamma ^\mu C = \epsilon ' C \gamma ^\mu \end{aligned}$$
$$\bullet $$
a self-adjoint operator *D* on ${\mathcal {H}}$ satisfying the *order-one condition*
  2.2 $$\begin{aligned} {\big [[ D_{\mathrm {f}},\varrho (a)], J_{\mathrm {f}}\varrho (b)J_{\mathrm {f}}^{-1}\big ]=0 \qquad \text{ for } \text{ all } a,b\in {\mathcal {A}}} \end{aligned}$$
$$\bullet $$
the condition^5^${D\gamma _{\mathrm {f}}= -\gamma _{\mathrm {f}}D}$ for even *s*. Moreover, the three signs above impose:
  2.3a $$\begin{aligned} J^2_{\mathrm {f}}&=\epsilon \, , \end{aligned}$$
  2.3b $$\begin{aligned} J_{\mathrm {f}}D_{\mathrm {f}}&=\epsilon ' D_{\mathrm {f}}J_{\mathrm {f}}, \end{aligned}$$
  2.3c $$\begin{aligned} J_{\mathrm {f}}\gamma _{\mathrm {f}}&=\epsilon '' \gamma _{\mathrm {f}}J_{\mathrm {f}}. \end{aligned}$$

Notice that, in this setting, the square of ${J_{\mathrm {f}}}$ is obtained from *C* as specified above, but we added the redundant Eq. (2.3a), as this equation appears so for general real, even spectral triples. For *s* odd, ${\gamma _{\mathrm {f}}}$ can be trivial ${\gamma _{\mathrm {f}}=1_{{\mathcal {H}}}}$. The number $d:=p+q$ is the *dimension* and $s:=q-p$ (mod 8) is the *KO-dimension*.

### Remark 2.2

It will be useful later to stress that the ‘commutant property’ (cf. for instance [57, eq. 4.3.1])

2.4 $$\begin{aligned} {[}a,J b^* J^{-1}] =0,\qquad \text {for all } a,b \in {\mathcal {A}}, \end{aligned}$$

which is typically an axiom for spectral triples, is not assumed in our setting. However, one can show that it is a consequence of those in Definition 2.1. The axiom states that the right ${\mathcal {A}}$-action $\psi b:=b^{\mathrm {o}} \psi = J b^* J^{-1}\psi$, for $b \in {\mathcal {A}}, \psi \in {\mathcal {H}}$, commutes with the left ${\mathcal {A}}$-action $\varrho$ for each $a,b\in {\mathcal {A}}$. Since $J=C\otimes *$, and the algebra acts trivially on *V*,

2.5 $$\begin{aligned} a b^{\mathrm {o}} (v\otimes m )&= a J ( v \otimes b^* m^* ) = v \otimes (a m b ) \\&= b^{\mathrm {o}} a (v\otimes m), \quad v \in V, m\in M_N({\mathbb {C}}). \nonumber \end{aligned}$$

The focus of this paper is dimension four, but we still proceed in general dimension. We impose on the gamma matrices $\gamma ^\mu$ the following conditions:

2.6a $$\begin{aligned} (\gamma ^\mu )^2&=+1_V, \text { and } \gamma ^\mu \text{ Hermitian } \text{ for } \mu =1,\ldots , p, \end{aligned}$$

2.6b $$\begin{aligned} (\gamma ^\mu )^2&=-1_V, \text { and } \gamma ^\mu \text{ anti-Hermitian } \text{ for } \mu =p+1,\ldots ,p+ q. \end{aligned}$$

Since it will be convenient to treat several signatures simultaneously, we let $(\gamma ^\mu )^2=: e_\mu 1_V$ for each $\mu =1,\ldots ,d$. According to Eq. (2.6), one thus obtains the unitarity of all gamma-matrices:

$$\begin{aligned} (\gamma ^\mu v,\gamma ^\mu w)= ( (\gamma ^\mu )^* \gamma ^\mu v, w) = (e_\mu \gamma ^\mu \gamma ^\mu v, w) =(e_\mu )^2 ( v, w)=( v, w) \end{aligned}$$

without implicit sum, and for each $v,w \in V$. Let these matrices generate $\Omega := \langle \gamma ^1,\ldots ,\gamma ^d \rangle _{\mathbb {R}}$ as algebra, for which one obtains a splitting $\Omega = \Omega ^+\oplus \Omega ^-$ where $\Omega ^\pm$ is contains products of even/odd number of gamma-matrices. According to [3, Eq. 64], the Dirac operator ${D_{\mathrm {f}}}$ solves the axioms of an even-dimensional fuzzy geometry whenever it has the next form:

2.7 $$\begin{aligned} D_{\mathrm {f}}(v\otimes T)&= \sum _{I } \gamma ^I v \otimes \{ K_I , T \}_{e_I} \text { and } e_I\in \{+1,-1\},\\ \{A,B\}_{\pm }&:= AB \pm BA ,\nonumber \end{aligned}$$

where $T\in M_N({\mathbb {C}})$ and the sum is over increasingly ordered multi-indices $I=(\mu _1,\ldots ,\mu _{2r-1})$ of odd length. With such multi-indices *I*, the following product $\gamma ^I:=\gamma ^{\mu _1}\cdots \gamma ^{\gamma _{2r-1}} \in \Omega ^-$ is associated (the sum terminates after finitely many terms, since gamma-matrices square to a sign times $1_V$). Moreover, still as part of the characterization of ${D_{\mathrm {f}}}$, $e_I$ denotes a sign chosen according to the following rules:

$$\bullet $$
if $\gamma ^I$ is anti-Hermitian (so $e_I=-1$), then $\{ K_I , T \}_{e_I}=[L_I,T]$, i.e.,  is a commutator of the anti-Hermitian matrix $K_I$ (denoted by $L_I$); and
$$\bullet $$
if $\gamma ^I$ is Hermitian, so must be $K_I$, which will be denoted by $H_I$. Then $e_I=+1$, and , so  is an anti-commutator with a Hermitian matrix $H_I$.

### Example

Some Dirac operators of fuzzy *d*-dimensional geometries, $d=2,3,4$ in several ‘types’ (or signatures) (*p*, *q*).

$$\bullet $$
*Type* (0,2). Then $s=d=2$, so $\epsilon '=1$. The gamma matrices are anti-Hermitian and satisfy $(\gamma ^i)^2=-1$. The Dirac operator is
$$\bullet $$
*Type* (0,3), $s=3$. In this signature, the gamma matrices can be replaced for the quaternion units ${\imath ,\jmath }$ and *k* to express the (0, 3)-geometry Dirac operator as^6^
$$\bullet $$
*Type* (0,4), $s=4$, *Riemannian*. Since the triple product of anti-Hermitian gamma matrices is self-adjoint, $(\gamma ^\alpha \gamma ^\mu \gamma ^\nu )^*=(-)^3\gamma ^\nu \gamma ^\mu \gamma ^\alpha =\gamma ^\alpha \gamma ^\mu \gamma ^\nu$, so are the operator-coefficients, which have then the form  for $(H_{\alpha \mu \nu })^*=H_{\alpha \mu \nu }$:  where $\gamma ^{{{\hat{\rho }}}}$ means the product of gamma matrices with indices different from $\rho$, multiplied in ascending order; see the restriction in the sum in the expression for ${D^{(0,4)}_{\mathrm {f}}}$.
$$\bullet $$
*Type* (1,3), $s=2$, *Lorentzian*. Let $\gamma ^0$ be the time-like gamma matrix, i.e., the only one squaring to $+1$. Then 2.8

In the sequel, we use $K_I$ generically for either $H_I$ or $L_I$, whose adjointness-type is then specified by the signature and by *I*. We also define the sign $e_{I}$ by $K_I ^*:= e_I K_I$, or equivalently by $(\gamma ^I)^*= e_I \gamma ^I$, for a multi-index *I*. In four dimensions, one has for triple indices $I={{\hat{\mu }}}$ [41, App. A]

2.9 $$\begin{aligned} e_{{{\hat{\mu }}}} = e_\mu (-1)^{q+1} 1\le \mu \le d=p+q=4, \text{ for } \text{ signature } (p,q). \end{aligned}$$

In summary, a fuzzy geometry of signature (*p*, *q*) has following objects:

$$\bullet $$
$${{\mathcal {A}}_{\mathrm {f}}=M_N({\mathbb {C}})}$$
$$\bullet $$
${{\mathcal {H}}_{\mathrm {f}}=V \otimes M_N({\mathbb {C}})}$, Hilbert–Schmidt inner product on $M_N({\mathbb {C}})$
$$\bullet $$
a representation of ${{\mathcal {A}}_{\mathrm {f}}}$ on ${{\mathcal {H}}_{\mathrm {f}}}$, $\varrho (a) (v\otimes T)= v\otimes a T$
$$\bullet $$
${D_{\mathrm {f}}}$ given by Eq. (2.7)
$$\bullet $$
${J_{\mathrm {f}}=C\otimes *}$ with *C* anti-linear satisfying Eq. (2.1)
$$\bullet $$
${\gamma _{\mathrm {f}}=\gamma \otimes 1_{M_N({\mathbb {C}})}}$, with $\gamma$ constructed from all $\gamma$-matrices; see Eq. (3.4) for $d=4$

Although next equation is well-known, we recall it due of its recurrent usefulness later. In any dimension and signature, it holds:2.10Each inscribed segment in the *chord diagrams* denotes an index-pairing between two indices labeling their ends, say $\lambda$ and $\theta$, which leads to $\eta ^{\lambda \theta }$; all the pairings of each diagram are then multiplied bearing a total sign corresponding to $(-1)$ to the number of simple chord crossings. This picture is helpful to compute traces of more gamma-matrices, but is not essential here; see [41] to see how the spectral action for fuzzy geometries was computed by associating with these chord diagrams noncommutative polynomials in the different matrix blocks $K_I$ composing the Dirac operator. Incidentally, notice that so far this chord diagram expansion is classical, unlike that treated by Yeats [58, §9], which appears in the context of Dyson–Schwinger equations.

## Toward a ‘Matrix Spin Geometry’

We restrict the discussion from now on to dimension four, leaving the geometry type (KO-dimension) unspecified. Next, we elaborate on the similarity of the fuzzy Dirac operator and the spin-connection part spanned by multi-indices, which has been sketched in [3, Sec. V §A] for $d=4$. The identification works only in dimensions four and, if ‘unreduced’ (cf. Footnote 6 above) also three. For higher dimensions, quintuple products appear; for lower ones, triple products are absent. Although it would be interesting to address each dimensionality separately, since the physically most interesting case is dimension 4, we stick to it.

### Remark 3.1

Since some generality might be useful for the future, or elsewhere (e.g., in a pure Clifford algebra context), even though we identify the geometric meaning only for the objects in Riemannian signature, we prove most results in general signature.

For a Riemannian spin manifold *M*, recall the local expression (on an open $U\subset M$) of the canonical Dirac operator on the spinor bundle ${\mathbb {S}}\rightarrow M$ for each section $\psi$ there,

3.1a $$\begin{aligned} (D_M \psi ) (x)&= \mathrm {i}\Gamma ^j(x) \nabla _j^S \psi (x), \text { for } x\in U\text { and }\psi \in \Gamma ^\infty (U,{\mathbb {S}}), \end{aligned}$$

3.1b $$\begin{aligned} \nabla _i^S&= \partial _i + \omega _i. \end{aligned}$$

The coefficients $\omega _i= \frac{1}{2} \omega _i^{\mu \nu } \gamma _{\mu \nu }$ of the spin connection $\nabla ^S$ (the lift of Levi-Civita connection) are here expressed with respect to a base $\gamma _{\mu \nu }= \frac{1}{4} [\gamma _\mu , \gamma _\nu ]$ that satisfies the ${\mathfrak {o}}(4)$ Lie algebra in the spin representation (see, e.g., [17, §11.4]). The gamma matrices with Greek indices (or ‘flat’) $\gamma ^\mu$ relate to the above $\Gamma ^i(x) = e^i_\mu \gamma ^\mu$ by means of *tetrads* $e^i_\mu (x)$. The coefficients $e_\mu ^i\in C^\infty (U)$, by definition, make of the set of fields $(E_\mu )_{\mu =0,1,2,3}= (e_\mu ^i \cdot \partial _i)_{\mu =0,1,2,3}$ an orthonormal basis of ${\mathfrak {X}}(U)$ with respect to the metric *g* of *M*, which is to say $g(E_\mu ,E_\nu )= \eta _{\mu \nu }$. Thus $\{\Gamma ^i(x), \Gamma ^j (x) \}=2g^{ij}(x)=2(g^{-1})_{ij}(x)$ for $x\in U$, but $\{\gamma ^\mu ,\gamma ^\nu \}=2\eta ^{\mu \nu }$. In contrast to the commutation relations that the elements of the coordinate base $\partial _i=\partial / \partial x^i$ satisfy, one generally has $[E_\mu ,E_\nu ]\ne 0$ for the non-coordinate base $E_0,\ldots , E_3$, also sometimes called *non-holonomic* [55, §4]. Notice that in the fuzzy setting only Greek indices appear.

*This, together with the fact that rather* $\eta ^{\mu \nu }$ *instead of* $g^{ij}$ *appears in the Clifford algebra, should* *not* *be interpreted at this stage as flatness. Instead, for fuzzy geometries the equivalent of a metric is encoded in the signature* $\eta =\mathrm {diag}(e_0,\ldots , e_3)$ *and* *in the matrices parametrizing the Dirac operator.*

In Riemannian signature, we rewrite^7^ (cf. Ex. 2) 3.2a3.2b Simultaneously (up to the trivial factor $1_V$), we identify the commutators  with $\mathrm {i}E _\mu =\mathrm {i}e_\mu ^j \partial _j$ and the coefficients of the triple gamma products  with the full anti-symmetrization $\frac{\mathrm {i}}{4} \omega _{[\mu | ik } e^i_{|\sigma } e^k_{\nu ]}$ of the spin connection coefficients in the three Greek indices. The triple products of gamma-matrices present in the Dirac operator (3.2) are the analogue of those in the spin connection appearing in $D_M= \mathrm {i}\gamma ^\mu ( E_\mu + e_\mu ^i \omega _i)$, here in the ‘flat’ (non-holonomic or non-coordinate) basis $E_0,\ldots ,E_3$. Altogether, ${\nabla ^{S}_{\mathrm {f}}}$ can be understood as the matrix spin connection.

We let $\Delta _4=\{0,1,2,3\}$ and denote by $\delta _{\mu \nu \alpha \sigma }$ the fully symmetric symbol with indices in $\Delta _4$, which is non-vanishing (and then equal to 1) if and only if the four indices are all different; equivalently, $\delta _{\mu \nu \alpha \sigma } = |\epsilon _{\mu \nu \alpha \sigma }|$, in terms of the (flat) Levi-Civita symbol $\epsilon$.

Remark on notation. Specially when dealing with fuzzy geometries, we sometimes do not use Einstein’s summation (traditional in differential geometry). We avoid raising and lowering indices as well, e.g., gamma matrices are presented only with upper indices. We set $k=(k_\mu )_{\mu \in \Delta _4}, K=(K_\mu )_{\mu \in \Delta _4}, x=(x_\mu )_{\mu \in \Delta _4}$, et cetera.

### Lemma 3.2

For any $\mu ,\nu \in \Delta _4=\{0,1,2,3\}$ the following relations are satisfied for any signature $\eta =\mathrm {diag}(e_0,e_1,e_2,e_3)$ in four dimensions:

3.3a $$\begin{aligned} \gamma ^\mu \gamma ^{{{\hat{\nu }}}}&= (-1)^{\mu }\left( \delta ^{\mu }_\nu \gamma ^0 \gamma ^1\gamma ^2\gamma ^3 +\mathrm {sgn}(\nu -\mu )\sum _{\alpha < \sigma } \delta _{\mu \nu \alpha \sigma } e_\mu \gamma ^\alpha \gamma ^\sigma \right) , \end{aligned}$$

3.3b $$\begin{aligned} \gamma ^{{{\hat{\mu }}}}\gamma ^\mu&= - \gamma ^\mu \gamma ^{{{\hat{\mu }}}} , \end{aligned}$$

3.3c $$\begin{aligned} \gamma ^{{{\hat{\nu }}}}\gamma ^\mu&= +\gamma ^\mu \gamma ^{{{\hat{\nu }}}} \qquad (\nu \ne \mu ) , \end{aligned}$$

3.3d $$\begin{aligned} \gamma ^{{{\hat{\mu }}} }\gamma ^{{{\hat{\nu }}} }&= (-1)^{1+|\mu -\nu |}\sum _{\rho ,\lambda }\frac{1}{2} \delta _{\mu \nu \lambda \rho }e_\lambda e_\rho \gamma ^\mu \gamma ^\nu -1_V \delta ^\mu _{\nu } \cdot e_\mu \cdot \det (\eta ) . \end{aligned}$$

This lemma is proven in “Appendix A.” Notice that in Eq. (3.3) the repeated indices $\mu ,\nu$ in the RHS are not summed (therefore the index-symmetry of $\delta _{\mu \nu \lambda \rho }$ with the antisymmetry of $\gamma ^\mu \gamma ^\nu$ does annihilate that term).

We now need the explicit form of the chirality ${\gamma _{\mathrm {f}}=\gamma \otimes 1_{M_N({\mathbb {C}})}}$, given by

3.4 $$\begin{aligned} \gamma = (-\mathrm {i})^{\frac{1}{2}(q-p)(q-p+1)} \gamma ^0\gamma ^1\gamma ^2\gamma ^3 =: \sigma (\eta ) \gamma ^0\gamma ^1\gamma ^2\gamma ^3 . \end{aligned}$$

This factor $\sigma (\eta )$ in $\gamma$ in front of the matrices is $-1,+\mathrm {i},+1,-\mathrm {i}$, for the signatures $(p,q)= (0,4), (1,3), (2,2),(3,1)$, respectively, corresponding to KO-dimensions $s=4,2,0,6$.

### Lemma 3.3

The square of the Dirac operator of a fuzzy geometry ${G_{\mathrm {f}}}$ of signature $\eta =\mathrm {diag}(e_0,\ldots ,e_3)$ is

3.5 $$\begin{aligned} D^2_{\mathrm {f}}&= \sum _{\mu ,\nu }1_V \otimes \eta ^{\mu \nu }k_\mu \circ k_\nu + \frac{1}{2}\gamma ^\mu \gamma ^\nu \otimes [k_\mu , k_\nu ]_\circ - \sum _\mu \det (\eta ) e_\mu 1_V\otimes x_\mu \circ x_\mu \nonumber \\&\quad + \sum _{\mu < \nu } t_{\mu \nu } \gamma ^{\mu } \gamma ^\nu \otimes [x_\mu ,x_\nu ]_{\circ } +\frac{1}{2}\sum _{\mu ,\nu ,\sigma ,\alpha } s_{\mu \nu \alpha \sigma } \cdot \gamma ^\alpha \gamma ^\sigma \otimes \{ x_\nu ,k_\mu \}_{\circ }\\&\quad +\frac{1}{\sigma (\eta )} \sum _{\mu } {(-1)^\mu } \gamma \otimes [x_\mu ,k_\mu ]_{\circ },\nonumber \end{aligned}$$

with the ‘commutator’ $[f,g]_\circ$ given by $f\circ g - g\circ f$ in terms of the composition $\circ$ of the following operators (which are themselves commutators or anti-commutators)3.6We defined also the (whenever non-vanishing) signs

3.7 $$\begin{aligned} s_{\mu \nu \alpha \sigma }&:= e_\mu (-1)^{\mu } \cdot \mathrm {sgn}(\nu -\mu )\cdot \mathrm {sgn}(\sigma -\alpha ) \cdot \delta _{\mu \nu \alpha \sigma }\in \{-1,0,+1\} , \end{aligned}$$

3.8 $$\begin{aligned} t_{\mu \nu }&:= \sum _{\lambda < \rho }(-1)^{1+|\mu -\nu |}\delta _{\mu \nu \lambda \rho }e_\lambda e_\rho \in \{-1,0,+1\}. \end{aligned}$$

### Proof

One straightforwardly finds ${D^2 _{\mathrm {f}}= ({\mathfrak {a}}+{\mathfrak {b}}+{\mathfrak {c}}+{\mathfrak {d}}+{\mathfrak {e}})(k,x)}$ with

3.9a $$\begin{aligned} {\mathfrak {a}}(k,x)&= \sum _{\mu ,\nu } \gamma ^\mu \gamma ^\nu \otimes (k_\mu \circ k_\nu ), \end{aligned}$$

3.9b $$\begin{aligned} {\mathfrak {b}}(k,x)&=\sum _\mu \gamma ^{{{\hat{\mu }}}}\gamma ^\mu \otimes (x_\mu \circ k_\mu )+ \gamma ^\mu \gamma ^{{{\hat{\mu }}}}\otimes ( k_\mu \circ x_\mu ) ,\end{aligned}$$

3.9c $$\begin{aligned} {\mathfrak {c}}(k,x)&=\sum _{\mu \ne \nu } \gamma ^{{{\hat{\mu }}}}\gamma ^\nu \otimes (x_\mu \circ k_\nu ) + \gamma ^\mu \gamma ^{{{\hat{\nu }}}}\otimes ( k_\mu \circ x_\nu ) , \end{aligned}$$

3.9d $$\begin{aligned} {\mathfrak {d}}(k,x)&=\sum _\mu \gamma ^{{{\hat{\mu }}}}\gamma ^{{{\hat{\mu }}}}\otimes (x_\mu \circ x_\mu ), \end{aligned}$$

3.9e $$\begin{aligned} {\mathfrak {e}}(k,x)&=\sum _{\mu \ne \nu } \gamma ^{{{\hat{\mu }}}}\gamma ^{{{\hat{\nu }}}}\otimes (x_\mu \circ x_\nu ). \end{aligned}$$

For the first term, one obtains

$$\begin{aligned} {\mathfrak {a}}(k,x)&= \sum _{\mu ,\nu } \gamma ^\mu \gamma ^\nu \otimes k_\mu \circ k_\nu \\&=\sum _{\mu ,\nu } \gamma ^\mu \gamma ^\nu \otimes \frac{1}{2} \Big ( k_\mu \circ k_\nu + k_\nu \circ k_\mu + [k_\mu , k_\nu ] _{\circ } \Big )\\&=\sum _{\mu ,\nu }\gamma ^\mu \gamma ^\nu \otimes \frac{1}{2} ( k_\mu \circ k_\nu ) + \Big (\eta ^{\mu \nu }1_V - \frac{1}{2}\gamma ^\nu \gamma ^\mu \Big ) \otimes \Big ( k_\nu \circ k_\mu + [k_\mu , k_\nu ]\Big ) \\&=\sum _{\mu ,\nu } 1_V \otimes \eta ^{\mu \nu }k_\mu \circ k_\nu - \frac{1}{2}\gamma ^\nu \gamma ^\mu \otimes [k_\mu , k_\nu ] _{\circ }\,. \end{aligned}$$

To get the first two terms in the RHS of Eq. (3.5), one renames indices in the last term. The third summand is precisely ${\mathfrak {d}}$ after applying Lemma 4.4 with $\mu =\nu$. The fourth term is ${\mathfrak {e}}$, also by Lemma 4.4. The sixth and last term in Eq. (3.5) comes from ${\mathfrak {b}}$; if one uses $\{\gamma ^{{{\hat{\mu }}}},\gamma ^\mu \}=0$ and Eq. (3.3a)$|_{\mu =\nu }$, after using introducing the chirality element:

$$\begin{aligned} {\mathfrak {b}}(k,x)&=\sum _\mu \gamma ^\mu \gamma ^{{{\hat{\mu }}}}\otimes ( k_\mu \circ x_\mu -x_\mu \circ k_\mu )\\&= \sum _\mu (-1)^\mu \gamma \otimes [k_\mu ,x_\mu ] \qquad (\text {via Eq.}~3.4). \end{aligned}$$

We now see that the only Gothic letter left unmatched, ${\mathfrak {c}}$, is precisely the fifth term. Indeed, due to Lemma 3.2,

$$\begin{aligned} {\mathfrak {c}}(k,x)&= \sum _{\mu \ne \nu } \gamma ^\nu \gamma ^{{{\hat{\mu }}}}\otimes (x_\mu \circ k_\nu ) + \gamma ^\mu \gamma ^{{{\hat{\nu }}}}\otimes ( k_\mu \circ x_\nu ) \quad \text {(by Eq.}~{\mathrm{3.3c}}) \\&= \sum _{\mu \ne \nu } \gamma ^\mu \gamma ^{{{\hat{\nu }}}}\otimes (x_\nu \circ k_\mu ) + \gamma ^\mu \gamma ^{{{\hat{\nu }}}}\otimes ( k_\mu \circ x_\nu ) \quad \text {(index renaming)} \\&= \sum _{\mu \ne \nu } \gamma ^\mu \gamma ^{{{\hat{\nu }}}}\otimes \{ x_\nu ,k_\mu \}\\&= \sum _{\mu \ne \nu } (-1)^{\mu } e_\mu \sum _{\alpha < \sigma } (\delta _{\mu \nu \alpha \sigma } \mathrm {sgn}(\nu - \mu ) )\gamma ^\alpha \gamma ^\sigma \otimes \{ x_\nu ,k_\mu \} \quad \text {(by Lemma}~3.2)\\&=\frac{1}{2} \sum _{\mu ,\nu ,\alpha ,\sigma } \big [ (-1)^{\mu } e_\mu \mathrm {sgn}(\nu - \mu ) \mathrm {sgn}(\alpha - \sigma )\delta _{\mu \nu \alpha \sigma } \big ]\gamma ^\alpha \gamma ^\sigma \otimes \{ x_\nu ,k_\mu \} \end{aligned}$$

where in the last step we exploited the skew-symmetry of the gammas with different indices to annul the restriction $\alpha < \sigma$ on the sum by introducing $\mathrm {sgn}(\sigma -\alpha )$. The term in square brackets is $s_{\mu \nu \alpha \sigma }$. $\square$

**Table 1:** Analogies between smooth spin geometry and Riemannian fuzzy geometries

Meaning	Random matrix case (Riemannian signature)	Smooth operator
Derivation		$\partial _i$
Gauge potential		$\mathbb A_i$
Higgs field	$\Phi$	*H*
Covariant Derivative		$\mathbb D_i=\partial _i + \mathbb A_i$
Field strength		$[\mathbb D_i, \mathbb D_j] = \overbrace{\,[\partial _i, \partial _j]\,}^{\,\,\,\,\equiv \,0}\! \,+$
		$$ \partial _i \mathbb A_j -\partial _j \mathbb A_i + [\mathbb A_i,\mathbb A_j]$$
Higgs lagrangian	${{\,\mathrm{Tr}\,}}(a_2\Phi ^2 + a_4 \Phi ^4 )$	$\int _M \big (a_2|H|^2 + a_4 |H|^4 \big )\mathrm {vol}$
Gauge-Higgs coupling		$-\int _M |\mathbb D_i H|^2\mathrm {vol}$
Yang-Mills action	$-\frac{1}{4} {{\,\mathrm{Tr}\,}}({\mathscr {F}}_{\mu \nu }{\mathscr {F}}^{\mu \nu })$	$-\frac{1}{4} \int _M {{\,\mathrm{Tr}\,}}_{\mathfrak {su}(n)} (\mathbb F_{ij} \mathbb F^{ij})\mathrm {vol}$

Local expressions in a chart *U* of *M* are given. Here, ${\mathfrak {X}}(U)$ are the vector fields on *U*, whose non-coordinate base is $\{E_\mu \}$

Notice that the analogy in Table 1 goes further, since in the case of a smooth manifold spin manifold (*M*, *g*), the fields $\partial _0,\ldots ,\partial _3$, or equivalently $E_0,\ldots , E_3$, (locally) span the space of vector fields ${\mathfrak {X}}(M)$ on *M*, that is, derivations in $C^\infty (M)$. The analogue of $\partial _j$ is here (after the base change to $E_\mu$) the derivation in $\mathrm {Der}(M_N({\mathbb {C}}))$ that corresponds to .

## Gauge Matrix Spectral Triples

We restrict the discussion to even KO-dimensions ($\epsilon '=1$) and define the main spectral triples for the rest of the article. Their terminology is inspired by the results. The reader might want to see Table 2, which will be hopefully helpful to grasp the organization of the objects introduced this section. But first, we recall that the spectral triple product $G_1\times G_2$ of two real, even spectral triples $G_i=({\mathcal {A}}_i,{\mathcal {H}}_i,D_i, J_i,\gamma _i)$ is

$$\begin{aligned} ({\mathcal {A}}_1\otimes {\mathcal {A}}_2 ,{\mathcal {H}}_1\otimes {\mathcal {H}}_2, D_1\otimes 1_{{\mathcal {H}}_2} + \gamma _1 \otimes D_2, J_1\otimes J_2, \gamma _1\otimes \gamma _2). \end{aligned}$$

### Definition 4.1

We define a *gauge matrix spectral triples* as the spectral triple product ${G_{\mathrm {f}}\times F}$ of a fuzzy geometry ${G_{\mathrm {f}}}$ with a finite geometry $F=({\mathcal {A}}_F,{\mathcal {H}}_F,D_F, J_F,\gamma _F)$, $\dim {\mathcal {A}}_F < \infty$. If *F* is a finite geometry with ${\mathcal {A}}_F=M_n({\mathbb {C}})$ and ${\mathcal {H}}_F=M_n({\mathbb {C}})$ with $2\le n$, we say that ${G_{\mathrm {f}}\times F}$ is a *Yang–Mills–Higgs matrix spectral triple*. If moreover $D_F=0$ above holds, then ${G_{\mathrm {f}}\times F}$ is called *Yang–Mills matrix spectral triple*.

We should denote these geometries by ${G^{(N)}_{\mathrm {f}}\times F^{(n)}}$, but for sake of a compact notation, we leave those integers implicit and write ${G_{\mathrm {f}}\times F}$.

### Yang–Mills Theory From Gauge Matrix Spectral Triples

In order to derive the SU(*n*)-Yang–Mills theory on a fuzzy base, we choose the following inner space algebra: ${\mathcal {A}}_F= M_n({\mathbb {C}})$. This algebra acts on the Hilbert space ${\mathcal {H}}_F=M_n({\mathbb {C}})$ by multiplication. The Connes’ 1-forms $\Omega ^1_{D}({\mathcal {A}})$ for ${\mathcal {A}}=M_N({\mathbb {C}})\otimes M_n({\mathbb {C}})$ are then elements of the form

4.1 $$\begin{aligned} \omega = \sum {{\mathsf {a}}}[D, {{\mathsf {c}}}] \, \,\,\mathrm {with} \,\,\, {{\mathsf {a}}}= \sum W \otimes a ,\,\,\, {{\mathsf {c}}}=\sum T \otimes c \in M_N({\mathbb {C}})\otimes M_n({\mathbb {C}}), \end{aligned}$$

where the sums are finite. The latter algebra is the fuzzy analogue of the algebra $C^\infty (M, {\mathcal {A}}_F)=C^\infty (M)\otimes {\mathcal {A}}_F$ of an ($\infty$-dimensional, smooth) almost-commutative geometry.

**Table 2:**
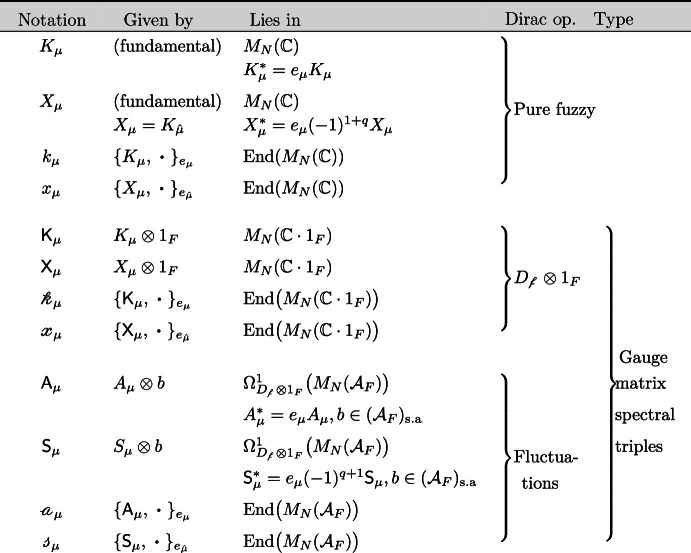
Notation for different matrices and operators appearing in the Dirac operator ${D=D_{\mathrm {f}}\otimes 1_F}$ of ${G_{\mathrm {f}}\times F}$ (case with $D_F=0$) and its fluctuations

In the table $M_N({\mathbb {C}}\cdot 1_F)=M_N({\mathbb {C}})\otimes ( {\mathbb {C}}\cdot 1_F)$

In order to compute the fluctuated Dirac operator, we start in this section with the fluctuations along the fuzzy geometry (labeled with ${\mathrm {f}}$) and leave those along the *F* direction for the Sect. 5. Thus, turning off the ‘finite part’ $D_F=0$, one obtains

4.2 $$\begin{aligned} {D_{{\mathrm{gauge}}}:=D_{\omega _{\mathrm {f}}}= D_{\mathrm {f}}\otimes 1_F + \omega _{\mathrm {f}}+ J \omega _{\mathrm {f}}J^{-1}} \end{aligned}$$

for ${\omega _{\mathrm {f}}}$ of the form (4.1), with respect to the ‘purely fuzzy’ Dirac operator 4.3a4.3b

#### Theorem 4.2

On the Yang–Mills matrix spectral triple over a four-dimensional fuzzy geometry of type (*p*, *q*), i.e., of signature $\eta =\mathrm {diag}(+_p,-_q)$, the fluctuated Dirac operator ${D=D_{\mathrm {f}}\otimes 1_F}$ reads4.4in terms of matrices ${{\mathsf {A}}_\mu , {\mathsf {S}}_\mu \in \Omega ^1 _D({\mathcal {A}}_{\mathrm {f}}\otimes {\mathcal {A}}_F)}$ satisfying

4.5 $$\begin{aligned} ( {\mathsf {A}}_\mu )^*&= e_\mu {\mathsf {A}}_\mu ,\qquad \text {and}\qquad ( {\mathsf {S}}_\mu )^*= (-1)^{q + 1 }e_\mu {\mathsf {S}}_\mu . \end{aligned}$$

Here, the curly brackets are a generalized commutator $\{A,B\}_{\pm } = AB \pm BA$ depending on $e_\mu ,e_{{{\hat{\mu }}}}\in \{+1,-1\}$.

#### Proof

The theorem follows by combination of Lemma 4.3 with Lemma 4.4, both proven below. $\square$

#### Lemma 4.3

(Fluctuations with respect to the $K_\mu$-matrices). With the same notation of Theorem 4.2 and setting $X_\mu = K_{{{\hat{\mu }}}}=0$—cf. Eq. (3.2) and Eq. (3.6)—the innerly fluctuated Dirac operator $D_{{\mathrm{gauge}}}$ is given by4.6

#### Proof of Lemma 4.3

We set $X=0$ globally in this proof. Pick a homogeneous vector in the full Hilbert space $\Psi =v\otimes Y\otimes \psi \in {\mathcal {H}}= V \otimes M_N({\mathbb {C}})\otimes {\mathcal {H}}_F$. For ${{\mathsf {a}}}=1_V\otimes W \otimes a$ and ${{\mathsf {a}}}'=1_V\otimes T \otimes c$ parametrized by $T,W\in M_N({\mathbb {C}})$ and $a,c\in {\mathcal {A}}_F$, the action of $\omega$ on $\Psi$ yieldsso ${\omega _{\mathrm {f}}= \sum _\mu \gamma ^\mu \otimes A_\mu \otimes b}$, relabeling $b=a c \in {\mathcal {A}}_F$ and $A_\mu : = W [K_\mu , T]$. Notice that since

4.7 $$\begin{aligned} (\gamma ^\mu \otimes A_\mu \otimes b)^*&= e_\mu \gamma ^\mu \otimes A_\mu ^* \otimes b^* \end{aligned}$$

the self-adjointness condition ${\omega _{\mathrm {f}}^*= \omega _{\mathrm {f}}}$ is achieved if and only if $(A_\mu \otimes b)^*=e_\mu (A_\mu \otimes b)$ for each $\mu$. The second part of the inner fluctuations is, for each

$$\begin{aligned} \Psi =v\otimes Y\otimes \psi \in V \otimes M_N({\mathbb {C}})\otimes M_n({\mathbb {C}}), \end{aligned}$$

the next expression:

$$\begin{aligned} (J \omega _{\mathrm {f}}J^{-1}) (\Psi )&= \big ( J {{\mathsf {a}}}[D_{\mathrm {f}}\otimes 1_F, {{\mathsf {a}}}' ] J^{-1}\big ) (\Psi ) \\&= \sum _\mu (C\otimes *_N \otimes *_n ) (\gamma ^\mu C^{-1}v \otimes A_\mu Y^* \otimes b \psi ^*)\\&= \sum _\mu (\underbrace{C \gamma ^\mu C^{-1}}_{\gamma ^\mu } v \otimes (A_\mu Y^*)^* \otimes (b \psi ^*)^* \qquad \text{(cf. } \text{ Eq. }~2.1)\\&= \sum _\mu \gamma ^\mu v \otimes YA_\mu ^* \otimes \psi b^*\\&= \sum _\mu ( \gamma ^\mu \otimes 1_{M_N({\mathbb {C}})}\otimes 1_n) \Psi (1_V\otimes A_\mu \otimes b)^*\\&= \sum _\mu (e_\mu \gamma ^\mu \otimes 1_{M_N({\mathbb {C}})}\otimes 1_n) \Psi (1_V\otimes A_\mu \otimes b), \end{aligned}$$

where the last step is a consequence of Eq. (4.7). Thus  where the bullet stands for the argument in $M_N({\mathbb {C}})\otimes M_n({\mathbb {C}})\subset {\mathcal {H}}$ to be multiplied by the right. Hence, 4.8a4.8b As a result, the fully-fluctuated operator acting on $\Psi =v \otimes Y\otimes \psi \in {\mathcal {H}}$ is

$$\begin{aligned} D_{\omega _{\mathrm {f}}} \Psi&= \overbrace{\sum _\mu \gamma ^\mu v \otimes \{K_\mu , Y\}_{ e_\mu } \otimes \psi }^{(D_{\mathrm {f}}\otimes 1_F) \Psi } + \sum _\mu \gamma ^\mu v \otimes \big ( A_\mu Y \otimes b \psi + e_\mu Y A_\mu \otimes \psi b\big ) . \end{aligned}$$

or defining ${\mathsf {K}}_\mu := K_\mu \otimes 1_n$ and ${\mathsf {A}}_\mu := A_\mu \otimes b \in M_N({\mathbb {C}})\otimes M_n({\mathbb {C}})$, one has$\square$

The triviality of the part of the Dirac operator along the finite geometry *F* implies that

$$\begin{aligned} {\Omega ^1_{D}({\mathcal {A}})= \Omega ^1 _{D_{\mathrm {f}}\otimes 1_F}\big ( M_{N\otimes n}({\mathbb {C}})\big )= \Omega ^1 _{D_{\mathrm {f}}}(M_N({\mathbb {C}})) \otimes M_n({\mathbb {C}}),} \end{aligned}$$

where $M_{N\otimes n}({\mathbb {C}})$ abbreviates $M_N({\mathbb {C}})\otimes M_n({\mathbb {C}})$ (in sub-indices, later further shortened as $M_{N\otimes n}^{{\mathbb {C}}}$ too), and the significance of each factor can be obtained by comparison with the smooth case. There, the inner fluctuations of a Dirac operator on an almost-commutative geometry are given by

$$\begin{aligned} \sum _\mu \Gamma ^\mu \otimes ({\mathbb {A}}_\mu - J_F {\mathbb {A}}_\mu J_F ),\,\,\,\mathrm {with} \,\,{\mathbb {A}} _\mu =- \mathrm {i}a \partial _\mu {b} \in C^\infty (M) \otimes {\mathcal {A}}_F\, . \end{aligned}$$

Recall that in the smooth case it is customary to treat only Riemannian signature together with self-adjointness (which we do not assume) for each gamma-matrix $\Gamma ^i= \mathrm {c}(\mathrm {d}x^i)$, $\mathrm {c}$ being Clifford multiplication. For each point *x* of the base manifold *M*, one has ${\mathbb {A}}_i(x)\in \mathrm {i}\, \mathfrak {su}(n)= \mathrm {i}\, \mathrm {Lie \,SU}(n)$. Since Eq. (4.8b) represents the fuzzy analogue, that equation can be further reduced to $b^*=b\in M_n({\mathbb {C}})_{\mathrm {s.a.}}=\mathrm {i}\,{\mathfrak {u}}(n)$ and $(A_\mu )^*=e_\mu A_\mu$, that is

4.9 $$\begin{aligned} A_\mu \in {\left\{ \begin{array}{ll} \mathrm {i}\,\mathfrak {u} (N) &{}\quad \text {if } e_\mu = +1 \text { iff } (\gamma ^\mu )^*= + \gamma ^\mu , \\ \mathfrak {u} (N) &{}\quad \text {if } e_\mu = -1 \text { iff } (\gamma ^\mu )^*= -\gamma ^\mu . \end{array}\right. } \end{aligned}$$

We now have to add the fluctuations resulting from the triple products of gamma matrices.

#### Lemma 4.4

(Fluctuations with respect to the $X_\mu$-matrices). With the same notation of Theorem 4.2 and additionally setting $K_\mu =0$, the innerly fluctuated Dirac operator $D_{{\mathrm{gauge}}}$ is given by4.10

#### Proof

See “Appendix A.” $$\square $$

From the last subsections, the rules for $S_\mu$ and $A_\mu$ lead to the manifest self-adjointness of ${D_{\omega _{\mathrm {f}}}}$.

### Field Strength and the Square of the Fluctuated Dirac Operator

We introduce now the main object of the gauge theory. To this end, let^8^$[{\mathsf {f}},{\mathsf {g}}]_{\circ }= ({\mathsf {f}}\circ {\mathsf {g}})-(\mathsf g\circ {\mathsf {f}})$ for any endomorphisms ${\mathsf {f}},{\mathsf {g}}$ of the same vector space. Similarly, we define $\{{\mathsf {f}},{\mathsf {g}}\}_\circ = ({\mathsf {f}}\circ {\mathsf {g}}) + (\mathsf g\circ {\mathsf {f}})$.

#### Definition 4.5

We abbreviate the following (anti)commutators 4.11a4.11b It follows in particular that . The *field strength* ${\mathscr {F}}_{\mu \nu }\in \mathrm {End} ( {\mathcal {A}}_{\mathrm {f}}\otimes {\mathcal {A}}_F)$ of a gauge matrix spectral triple ${G_\mathrm {f}\times F}$ is defined as4.12where .

#### Proposition 4.6

The square of the fluctuated Dirac operator of a Yang–Mills gauge matrix spectral triple that is flat ($X=0$, ) is given by

4.13 $$\begin{aligned} D_{{\mathrm{gauge}}}^2|_{X=0}= \frac{1}{2} \sum _{\mu ,\nu }\gamma ^\mu \gamma ^\nu \otimes {\mathscr {F}}_{\mu \nu }+ 1_V\otimes \vartheta , \end{aligned}$$

where4.14

#### Proof

Squaring ${D_{{\mathrm{gauge}}}= D_{\mathrm {f}}\otimes 1_F +\omega _{\mathrm {f}}+ J\omega _{\mathrm {f}}J^{-1}}$ one gets

4.15 $$\begin{aligned} D_{{\mathrm{gauge}}}^2&= (D_{\mathrm {f}})^2 \otimes 1_F + (D_{\mathrm {f}}\otimes 1_F )(\omega + J\omega J^{-1}) \\&\quad + (\omega + J\omega J^{-1}) (D_{\mathrm {f}}\otimes 1_F) + (\omega + J\omega J^{-1}) ^2 . \nonumber \end{aligned}$$

The first summand is known from Lemma 3.3. One obtains the last summand by Eq. (4.6) and using the Clifford algebra relations just as in the proof of that lemma. The result reads4.16being $[f,g]=f\circ g - g\circ f$ a simplified notation for the composition-commutator. We renamed indices and rewrote the last summand in (4.16) as  To make the notation lighter, we also mean by  the composition  from now on (also for ). Using Eq. (4.6), one can obtain for the two summands in the middle of Eq. (4.15); further abbreviating  one obtains4.17Again, we used the Clifford relations for the gamma matrices and renamed indices. Equations (4.16) and (4.17) implyExpanding Eq. (4.12)4.18and using Lemma 3.3$|_{X=0}$ together with  one gets the result.$\square$

#### Proposition 4.7

The fluctuated Dirac operator of a finite Yang–Mills geometry satisfies4.19

#### Proof

According to Eq. (4.4)so $D_\omega ^2$ has the same structure already observed in the ‘fuzzy Lichnerowicz formula’ above (Lemma 3.3). To be precise, notice that one can compute the square of the present Dirac operator by replacing the in ${D_{\mathrm {f}}}$ the following operators:  and . $\square$

### Gauge Group and Gauge Transformations

For any even spectral triple, the Hilbert space ${\mathcal {H}}$ is an ${\mathcal {A}}$-bimodule. The right action of ${\mathcal {A}}$ on the Hilbert space ${\mathcal {H}} \ni \Psi$ is implemented by the real structure *J*, $\Psi a:= a^{\mathrm {o}} \Psi := J a^* J^{-1}\Psi$. Both actions define the adjoint action $\mathrm {Ad}(u) \Psi := u \Psi u^*$ of the unitarities $u\in {\mathcal {U}}({\mathcal {A}})$ on ${\mathcal {H}}$. We want to determine the action of the unitarities $\mathcal {U} ({\mathcal {A}})=\{u \in {\mathcal {A}}\mid u^*u=1=uu^* \}$ of the algebra ${\mathcal {A}}$ on the Dirac operator,

4.20a $$\begin{aligned}&U \big (D + \omega + \epsilon ' J \omega J^{-1}\big ) U^* = D + \omega _u + \epsilon ' J \omega _u J^{-1}, \end{aligned}$$

4.20b $$\begin{aligned}&\quad U:=\mathrm {Ad}_u,\, u\in \mathcal {U}({\mathcal {A}}), \end{aligned}$$

which namely leads to the transformation rule

4.20c $$\begin{aligned} \omega \mapsto \omega _u = u \omega u^* +u[D,u^*] \end{aligned}$$

for the inner fluctuations. It is instructive to present a variation of the original proof given in [17, Prop. 1.141] for the analogous property of general spectral triples. Verifying this again is important, since the axiom $[a,b^{\mathrm {o}}]=0$ that appears in *op. cit.*, does not appear in the present axioms. However, according to Remark 2.2, it is a consequence of these in the fuzzy setting. So one can see that not only there, but also for gauge matrix spectral triples, the commutant property $[{\mathcal {A}},{\mathcal {A}}^{\mathrm {o}}]=0$ (elsewhere an axiom) holds. Indeed, since $J=C\otimes *_N \otimes *_n$, for $a,b\in {\mathcal {A}}$,

4.21 $$\begin{aligned} a b^{\mathrm {o}} (v\otimes m ) = a J ( v \otimes b^* m^* )&= v \otimes (a m b )\\&= b^{\mathrm {o}} a (v\otimes m), \quad v \in V, m\in M_{N\otimes n}({\mathbb {C}}). \nonumber \end{aligned}$$

The commutant property is essential for the subalgebra

4.22 $$\begin{aligned} {\mathcal {A}}_{J}:=\{ a \in {\mathcal {A}}\mid a J = J a^*\} \subset {\mathcal {A}}\end{aligned}$$

to be also a subalgebra of the center $Z({\mathcal {A}})$, as we will see later.

*Proof of* Eq. (4.20)*; adapted from* [17, §10] *to fuzzy geometries* We split the adjoint action into the right action by $u^*$, $z:=(u^*)^{\mathrm {o}}=J u J^{-1}$, and the left action by *u*, $U=u z$.

$$\bullet $$
Transformation of *D*: Applying $wD w^* = D + w [D,w^*]$ consecutively for $w=z,u$, one gets
  4.23 $$\begin{aligned} U D U^* = u ( D + z[D,z]) u^* = D + u[D,u^*] + z[D,z^*]. \end{aligned}$$
$$\bullet $$
Transformation of $\omega$: since $\omega \in \Omega ^1_D({\mathcal {A}})$, $\omega = a[D,b]$ (or sum of this 1-forms), one also has
  $$\begin{aligned} \omega z^* = \omega u^{\mathrm {o}} {\mathop {=}\limits ^{2.2}} a[D,b] u^{\mathrm {o}} =a u^{\mathrm {o}}[D,b] {\mathop {=}\limits ^{2.5}} u^{\mathrm {o}} a [D,b] = z^* \omega , \end{aligned}$$
  so $$ U \omega U^* = u (z \omega z ^*)u = u \omega u^* $$, since $$z z ^*=1$$. Also the term $$u[D,u^*] $$ is absorbed from the pure Dirac operator, then
  $$\begin{aligned} \omega \mapsto u \omega u^* + u[D,u^*]. \end{aligned}$$
$$\bullet $$
Transformation of $J\omega J^{-1}$: Similarly one obtains $U J \omega J^{-1}U^* \!=\! J (u \omega u^* ) J$. But actually $z[D,z^*]$ from Eq. (4.23) can be taken from the transformation of the pure Dirac operator and passed to that of $J \omega J^{-1}$, contributing, by the axioms (2.3) of the fuzzy geometry, since one can rearrange it as
  $$\begin{aligned} z[D,z^*] = J u J^{-1}\big ( D J u^* J^{-1}- J u^* J^{-1}D \big )= \epsilon ' J u [D, u^*] J^{-1}. \end{aligned}$$

$$\square $$

The gauge group ${\mathsf {G}}$ of a real spectral triple is defined via the adjoint action $\mathrm {Ad}_u(a) = u a u^*$ of the unitary group $\mathcal {U}({\mathcal {A}})$ on ${\mathcal {H}}$ as follows:

4.24 $$\begin{aligned} {\mathsf {G}}({\mathcal {A}},J) = \{ \mathrm {Ad}_u \,|\, u\in \mathcal {U}({\mathcal {A}}) \} = \{ uJ uJ^{-1}\,| \,u \in \mathcal {U}({\mathcal {A}}) \}. \end{aligned}$$

Before proceeding to compute it for a case concerning our study, we do the notation more symmetric, setting $n_1=N$ and $n_2=n$ for the rest of this section. We assume $n_1 > n_2 \ge 2$. The next statement is not surprising, but due to the presence of the tensor product, some care is needed.

#### Proposition 4.8

Let ${G_1\times G_2=G_{\mathrm {f}}\times F}$ be a gauge matrix geometry, with algebra ${\mathcal {A}}=A_1\otimes A_2$, $A_1=M_{n_1}({\mathbb {C}})$ and $A_2 = M_{n_2}({\mathbb {C}})$, and reality $J=J_1\otimes J_2$. The gauge group is given by the product of unitary projective groups ${\mathsf {G}}({\mathcal {A}},J) = \mathrm {PU}(n_1) \times \mathrm {PU}(n_2)$.

Before proving this proposition, broken down in some lemmata below, we recall the characterization of the gauge group that will be used. Namely, the next short sequence is exact, according to [57, Prop. 6.5]:

4.25 $$\begin{aligned} 1\rightarrow \mathcal {U}({\mathcal {A}}_J) \rightarrow \mathcal {U} ({\mathcal {A}}) \rightarrow {\mathsf {G}}({\mathcal {A}},J)\rightarrow 1. \end{aligned}$$

Thus, if the groups $\mathcal {U}(Z({\mathcal {A}}))$ and $\mathcal {U}({\mathcal {A}}_J)$ coincide, then

4.26 $$\begin{aligned} {\mathsf {G}}({\mathcal {A}},J) \cong \mathcal {U}({\mathcal {A}}) / \mathcal {U}(Z({\mathcal {A}})) . \end{aligned}$$

We now verify that they do, so that after computing $\mathcal {U}({\mathcal {A}})$ and $\mathcal {U}(Z({\mathcal {A}}))$, we can finally obtain the gauge group by this isomorphism (4.26).

#### Lemma 4.9

For ${\mathcal {A}}$ and *J* as in Proposition 4.8, $\mathcal {U}(Z({\mathcal {A}})) =\mathcal {U}({\mathcal {A}}_J)$.

#### Proof

First, observe that if $a\in {\mathcal {A}}_J$ and $b \in {\mathcal {A}}$, then

$$\begin{aligned} a b= Ja^* J^{-1}b= a^{\mathrm {0} }b = b a^{\mathrm {0} } = b Ja^* J= b a, \end{aligned}$$

where one gets the equalities at the very left or very right by the defining property (4.22) of ${\mathcal {A}}_J$, and the third equality by the commutant property (4.21). Hence, ${\mathcal {A}}_J \subset Z({\mathcal {A}})$, and thus $\mathcal {U}({\mathcal {A}}_J) \subset \mathcal {U}(Z({\mathcal {A}}))$.

We only have to prove the containment $$\mathcal {U}(Z({\mathcal {A}})) \subset \mathcal {U}({\mathcal {A}}_J)$$. According to Lemma A.1 (proven in “Appendix A”), $$Z({\mathcal {A}})=Z(A_1\otimes A_2)= Z(A_1)\otimes Z (A_2)$$. Since the representation $$\varrho $$ of $$ A_1 \otimes A_2 $$ on $$ {\mathcal {H}}_1\otimes {\mathcal {H}}_2=V\otimes A_1 \otimes A_2$$ is the fundamental on each factor (except the trivial action on spinor space factor *V*) by Schur’s Lemma, each $$ Z(A_i)$$ consists of multiples of the identity. Then, for any $$z_1\otimes z_2 \in Z(A_1)\otimes Z(A_2)$$ one has

$$\begin{aligned} \varrho [( z_1\otimes z_2)^* ]J \Psi&= (1_V\otimes {{\bar{z}}}_1\otimes {{\bar{z}}}_2) (C\otimes *_1\otimes *_2) \Psi \\&= (C\otimes *_1\otimes *_2) (1 _V\otimes z_1\otimes z_2 )\Psi =J \varrho (z_1\otimes z_2 )\Psi \end{aligned}$$

where $*_i$ is the involution of $A_i$ and $\Psi$ an arbitrary vector in the Hilbert space described above. Therefore, $z_1\otimes z_2 \in {\mathcal {A}}_J$. One verifies that this proof leads equally to $Z({\mathcal {A}}) \subset {\mathcal {A}}_J$ by taking other representing element $z_1\lambda \otimes z_2 \lambda ^{-1}$ $(\lambda \in {\mathbb {C}}^\times )$ the same conclusion $Z({\mathcal {A}}) \subset {\mathcal {A}}_J$ is reached, which restricted to the unitarities gives $\mathcal {U}(Z({\mathcal {A}})) \subset \mathcal {U}({\mathcal {A}}_J)$. $\square$

#### Lemma 4.10

The following is a short exact sequence of groups:

$$\begin{aligned} 1 \rightarrow {\mathbb {C}}^\times \rightarrow \{{\mathbb {R}}^+ \times \mathrm {U} (n_1)\} \times _{|\det |} \{{\mathbb {R}}^+ \times \mathrm {U}(n_2)\} {\mathop {\rightarrow }\limits ^{\alpha }} \mathcal {U} (A_1\otimes A_2) \rightarrow 1, \end{aligned}$$

where^9^

4.27 $$\begin{aligned}&\{{\mathbb {R}}^+ \times \mathrm {U} (n_1)\} \times _{|\det |} \{{\mathbb {R}}^+ \times \mathrm {U}(n_2)\}\\&\quad := \big \{ (\rho _1,u_1,\rho _2,u_2) \in {\mathbb {R}}^+ \times \mathrm {U}(n_1) \times {\mathbb {R}}^+ \times \mathrm {U}(n_2) \mid \rho _1\rho _2 =1 \big \}. \nonumber \end{aligned}$$

#### Proof

Let us abbreviate the group in the middle as follows $G= \{{\mathbb {R}}^+ \times \mathrm {U} (n_1)\} \times _{|\det |} \{{\mathbb {R}}^+ \times \mathrm {U}(n_2)\}$ and define $\alpha : G\rightarrow \mathcal {U} (A_1\otimes A_2)$ by $(\rho _1,u_1,\rho _2,u_2) \mapsto \rho _1u_1\otimes \rho _2u_2$. Suppose $(\rho _1,u_1,\rho _2,u_2)\in \ker \alpha$, so that $\alpha (\rho _1,u_1,\rho _2,u_2) =\rho _1 u_1\otimes \rho _2 u_2 =1_{n_1} \otimes 1_{n_2}$. Since $\ker \alpha \subset G$ one has $\rho _1\rho _2=1$. Thus, the previous equation yields $u_1\otimes u_2=1_{n_1} \otimes 1_{n_2}$, which says that in the lhs $u_1$ and $u_2$ are a scalar multiples and mutual inverses. Then $\ker \alpha \cong \{\rho ,\lambda ,\rho ^{-1},\lambda ^{-1}\}$, and if one embeds ${\mathbb {C}}^\times \hookrightarrow G$ as follows (which will be the definition of the leftmost map) $z=|z|\cdot \mathrm {e}^{\mathrm {i}\theta }= r \cdot \mathrm {e}^{\mathrm {i}\theta } \mapsto (r, \mathrm {e}^{\mathrm {i}\theta } ,r^{-1}, \mathrm {e}^{-\mathrm {i}\theta } )$, one gets exactness at *G*.

**Fig. 3:**
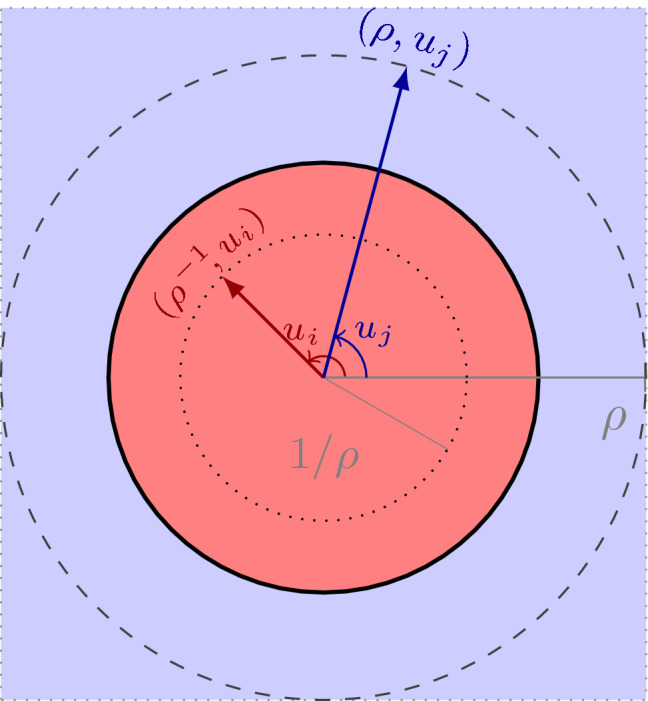
Illustration of the group *G*. Such group appears in the description of ${\mathcal {U}}(A_1\otimes A_2)\cong G / {\mathbb {C}}^\times$. There the indices refer to each $\mathrm {U}(n_i)$-factor, $i\ne j$, and the $\rho$ and $\rho ^{-1}$ might lie outside the unit circle (thick line)

The rightmost map $\mathcal {U} (A_1\otimes A_2) \rightarrow 1$ is the determinant in absolute value. Its kernel has elements $g_1\otimes g_2 \in \mathcal {U} (A_1\otimes A_2)$ satisfying $|\det ( g_1\otimes g_2)|=1$. But this condition is satisfied by all elements $g_i= \rho _i u_i$, as far as $(\rho _1,u_1,\rho _2,u_2 )\in G$. Conversely, if $g_1\otimes g_2 \in \mathcal {U} (A_1\otimes A_2)$ satisfies $|\det (g_1\otimes g_2)|=1$, then there exists a $\lambda = |\lambda | \cdot \mathrm {e}^{\mathrm {i}\psi } \in {\mathbb {C}}^\times$ with $g_1= \lambda \cdot u_1$ and $g_2=\lambda ^{-1}\cdot u_2$. Then $\alpha (|\lambda |, \mathrm {e}^{\mathrm {i}\psi } \cdot u_1 , |\lambda |^{-1}, \mathrm {e}^{\mathrm {i}\psi } \cdot u_2) =g_1\otimes g_2$. Hence,  too, and the sequence is exact also at $\mathcal {U} (A_1\otimes A_2)$ (Fig. 3). $\square$

#### Lemma 4.11

The following group sequence is exact:

$$\begin{aligned} 1\rightarrow {\mathbb {C}}^\times \rightarrow {\mathbb {C}}^\times \times _{|\det |} {\mathbb {C}}^\times \rightarrow \mathcal {U} \{Z(A_1\otimes A_2)\} \rightarrow 1. \end{aligned}$$

The group ${\mathbb {C}}^\times \times _{|\det |} {\mathbb {C}}^\times$ in the middle is the subgroup of $({\mathbb {C}}^\times )^2$ whose entries $(z_1,z_2)$ satisfy $|z_1|=|z_2|^{-1}$.

#### Proof

The embedding ${\mathbb {C}}^\times \hookrightarrow {\mathbb {C}}^\times \times _{|\det |} {\mathbb {C}}^\times$ is given by $\lambda \mapsto (\lambda ,\lambda ^{-1})$ and the next map ${\mathbb {C}}^\times \times _{|\det |} {\mathbb {C}}^\times \rightarrow \mathcal {U} \{Z(A_1\otimes A_2)\}$ by $(z_1,z_2) \mapsto z_1\otimes z_2$. Being the rest an easier case than that the proof of Lemma 4.10, the details on exactness can be deduced from there. $\square$

We are now in position to give the missing proof.

#### Proof of Proposition 4.8

According to Eq. (4.26),

$$\begin{aligned} {\mathsf {G}}({\mathcal {A}},J)&\cong {\mathcal {U} (A_1\otimes A_2)}\big /{ \mathcal {U} \{Z(A_1\otimes A_2)\}} \\&\cong {\mathcal {U} (A_1\otimes A_2)}\big /{ \mathcal {U} \{Z(A_1)\otimes Z(A_2)\}}. \end{aligned}$$

where one passes to the second line by Lemma A.1. By Lemma 4.11 for the group in the ‘numerator’ and Lemma 4.10 for the one in the ‘denominator,’

$$\begin{aligned} {\mathsf {G}}({\mathcal {A}},J)\cong \frac{ \big [\{{\mathbb {R}}^+ \times \mathrm {U} (n_1)\} \times _{|\det |} \{{\mathbb {R}}^+ \times \mathrm {U}(n_2)\}\big ]\big /{\mathbb {C}}^\times }{ \big ({\mathbb {C}}^\times \times _{|\det |} {\mathbb {C}}^\times \big ) /{\mathbb {C}}^\times } . \end{aligned}$$

By the third group isomorphism theorem, one can ‘cancel out’ the ${\mathbb {C}}^\times$, and get

$$\begin{aligned} {\mathsf {G}}({\mathcal {A}},J)\cong \frac{ \big [\{{\mathbb {R}}^+ \times \mathrm {U} (n_1)\} \times _{|\det |} \{{\mathbb {R}}^+ \times \mathrm {U}(n_2)\}\big ] }{ \big ({\mathbb {C}}^\times \times _{|\det |} {\mathbb {C}}^\times \big ) } . \end{aligned}$$

Notice that in each group $|\det |$ only constrains the real parts, while it respects the $\mathrm {U}(n_1)$ and $\mathrm {U}(n_2)$ in the numerator and the two factors $\mathrm {U}(1)$ of each ${\mathbb {C}}^\times$ in the denominator. We conclude that

$$\begin{aligned} {\mathsf {G}}({\mathcal {A}},J)\cong \frac{ \mathrm {U}(n_1)\times \mathrm {U}(n_2) }{\mathrm {U}(1)\times \mathrm {U}(1) }\cong \mathrm {PU}(n_1)\times \mathrm {PU}(n_2) . \end{aligned}$$

$$\square $$

### Unimodularity and the Gauge Group

It turns out that for real algebras the gauge group does not automatically include the unimodularity condition, and this property needs to be added by hand. Since this is relevant for the algebra that one uses as input to derive the Standard Model (cf. discussion in [57, Ch. 8.1.1, Ch. 11.2]) we address also the unimodularity of the gauge group, when the base itself is noncommutative.

Given a matrix representation $\varrho$ of a unital $*$-algebra *A*, the special unitary group of *A* is defined by

$$\begin{aligned} \mathcal {SU} (A ) := \{ m \in {\mathcal {U}}(A)\mid \det [\varrho (m)] =1 \} . \end{aligned}$$

We now define the following morphisms $\delta _i : \mathrm {GL}(n_i) \rightarrow {\mathbb {C}}^\times$,

4.28 $$\begin{aligned} \delta _1 (g_1)= [\textstyle \det _{n_1}(g_1)]^{n_2} \qquad \text { and } \qquad \delta _2(g_2) = [\det _{n_2}(g_2)]^{-n_1}, \end{aligned}$$

which shall be useful in the description of the special unitary group we care about (notice that both morphisms depend on the pair $(n_1,n_2)$ and the different signs in the exponents).

#### Lemma 4.12

The special unitary group of $A_1\otimes A_2$,

$$\begin{aligned} \mathcal {SU}(A_1\otimes A_2) = \{ u_1\otimes u_2 \in \mathcal {U}(A_1\otimes A_2) \mid \det (u_1\otimes u_2)=1 \} \end{aligned}$$

fits in a short exact sequence of groups:

$$\begin{aligned} 1 \rightarrow \mathrm {U}(1) \hookrightarrow \mathrm {U}(n_1) \times _{\det } \mathrm {U}(n_2 ) {\mathop {\rightarrow }\limits ^{\kappa }}\mathcal {SU}(A_1\otimes A_2) \rightarrow 1, \end{aligned}$$

where $\mathrm {U}(n_1) \times _{\det } \mathrm {U}(n_2 )$ is the (categorical) pullback of any of the two morphisms (4.28) along the remaining one.

#### Proof

Define the homomorphism $\kappa$ by $(u_1,u_2)\mapsto u_1\otimes u_2$. Suppose that $u_1 \otimes u_2 \in \ker \kappa$, so $\kappa (u_1,u_2) = u_1 \otimes u_2 = 1_{n_1}\otimes 1_{n_2}$. This means that there exists a $\lambda \in {\mathbb {C}}^\times$ with $u_1= \lambda 1_{n_1}$ and $u_2 = \lambda ^{-1}1_{n_2}$, but by assumption $u_i \in \mathrm {U}(n_i)$, so $\lambda \in \mathrm {U}(1)$. Thus, the image of the inclusion $\mathrm {U}(1) \hookrightarrow \mathrm {U}(n_1) \times _{\det } \mathrm {U}(n_2 )$ $\lambda \mapsto (\lambda 1_{n_1} ,\lambda ^{-1}1_{n_2})$ is the kernel of $\kappa$.

The last map to the right is the determinant. If $u_1 \otimes u_2 \in {{\,\mathrm{im}\,}}\kappa$, then by definition of the fibered group $\mathrm {U}(n_1) \times _{\det } \mathrm {U}(n_2 )$, $\delta _1 (u_1) = \delta _2(u_2)$ holds. But this happens if and only if $1=[\det _{n_1}(u_1)]^{n_2}\cdot [\det _{n_2}(u_2)]^{n_1}= \det (u_1\otimes u_2)=(\det \circ \kappa ) (u_1,u_2)$. Therefore, the image of $\kappa$ is in the kernel of the determinant.

On the other hand, if $g_1\otimes g_2\in \ker (\det ) \subset \mathcal {SU}(A_1\otimes A_2)$ then each $g_i \in \mathrm {GL}(n_i)$ (otherwise its determinant vanishes and by assumption it is 1) so we can write them in matrix polar form $g_i= p_i u_i$ with $u_i \in \mathrm {U}(n_i)$ and $p_i =p_i^*$ positive definite. Since, in particular, $p_1 u_1\otimes p_2u_2\in \mathcal {U}(A_1\otimes A_2)$, one obtains

4.29 $$\begin{aligned} 1_{n_1} \otimes 1_{n_2}= p_1 u_1 u_1^* p_1^* \otimes p_2 u_2 u_2^* p_2^* = p_1^2 \otimes p_2^2. \end{aligned}$$

Being both $p_i$’s positive definite Hermitian matrices, they can be written as $p_i= v_i \Lambda _i v_i^*$ for $\Lambda _i =\mathrm {diag}(\lambda _{i,1},\ldots , \lambda _{i,n})$ with $\lambda _{i,m} \ge 0$ and $v_i \in \mathrm {U} (n_i)$. But then Eq. (4.29) means the existence of certain $r\in {\mathbb {R}}^+$ for which $v_1 (\Lambda _1)^2 v_1^* = r \cdot 1_{n_1}$ and $v_2(\Lambda _2)^2 v_2^* = r^{-1}\cdot 1_{n_2}$. Solving each equation leads to $\Lambda _1=r^{1/2}1_{n_2}$ and $\Lambda _2 = r^{-1/2}1_{n_2}$, so we can forget the $v_i$’s, since $\Lambda _i$ is central.

In summary, there exist scalars $\rho _i$ such that $p_i=\rho _i 1_{n_i}$ with $\rho _i>0$ and $\rho _1=1/\rho _2$. This relation shows that $g_1 \otimes g_2 = u_1\otimes u_2 = \kappa (u_1,u_2)$, since in the tensor product $g_1 \otimes g_2 = ( \lambda ^{-1}g_1 ) \otimes (\lambda g_2)$ for any $\lambda \in {\mathbb {C}}^\times$ (here, in particular, choosing $\lambda = \rho _1$). By construction, $u_i$ are unitarities, which, by assumption, moreover satisfy $1=\det (g_1\otimes g_2) =\det ( u_1 \otimes u_2 )=\delta _1(u_1) / \delta _2(u_2)$. Hence, $(u_1,u_2)\in \mathrm {U}(n_1) \times _{\det } \mathrm {U}(n_2 )$ and $g_1\otimes g_2 = \kappa (u_1,u_2)$, which concludes the proof of exactness at $\mathcal {SU}(A_1\otimes A_2)$. $\square$

#### Lemma 4.13

The following sequence of groups is exact:

$$\begin{aligned} 1 \rightarrow \mu _{\mathrm {mcd}(n_1,n_2)} {\mathop {\rightarrow }\limits ^{\iota }}\mathrm {U}(1) \times \mathrm {SU}(n_1) \times \mathrm {SU}(n_2) {\mathop {\rightarrow }\limits ^{\xi }}\mathrm {U}(n_1)\times _{\det } \mathrm {U}(n_2) {\mathop {\rightarrow }\limits ^{\zeta }}\mu _{n_1\cdot n_2} \rightarrow 1 \end{aligned}$$

where $$\mathrm {mcd}(n_1,n_2)$$ is the maximum common divisor of $$n_1 $$ and $$n_2$$.

#### Proof

From left to right, we start defining the maps and checking exactness along the way. The first map is $\iota (\lambda ) = (\lambda , \lambda ^{-1}\cdot 1_{n_1}, \lambda \cdot 1_{n_2})$. Since $\det _{n_i}(z\cdot 1_{n_i})=z^{n_i}=1$ for $z \in \mu _{\mathrm {mcd}(n_1,n_2)}$, the map is well-defined, and clearly is also injective.

The next map is given by $\xi (z,m_1,m_2) = (z m_1,z^{-1}m_2)$. Since $m_i$ have unit determinant, the condition $\delta _1 (z m_1 ) = z = 1/ \delta _2(z^{-1}m_2)$ is satisfied (cf. Eq. (4.28) above). The pair $(z m_1,z^{-1}m_2)$ is thus in the fibered product $\mathrm {U}(n_1)\times _{\det } \mathrm {U}(n_2)$, by its definition and $\xi$ is thus well-defined.

To verify the exactness, notice that if $$(z,m_1,m_2) $$ is such that $$\xi (z,m_1,m_2)=(zm_1,z^{-1}m_2)=(1_{n_1},1_{n_2})$$, since each $$m_i \in \mathrm {SU}(n_i)$$, one has $$\det _{n_1}(z^{-1}1_{n_1})=1$$ and $$\det _{n_2}(z 1_{n_2})=1$$. Hence $$z \in \mu _{n_1}\cap \mu _{n_2}$$. Thus, $$(z,m_1,m_2)= (z,z^{-1}\cdot 1_{n_1}, z\cdot 1_{n_1})$$ and therefore $$\ker \xi \subset {{\,\mathrm{im}\,}}\iota $$ since the group that generates this intersection $$z \in \mu _{n_1}\cap \mu _{n_2}$$ is $$ \mu _{\mathrm {mcd}(n_1,n_2)}$$. The other containment holds also, since

$$\begin{aligned} \xi \circ \iota (\lambda )= \xi (\lambda , \lambda ^{-1}\cdot 1_{n_1}, \lambda \cdot 1_{n_1})= (\lambda \cdot [\lambda ^{-1}\cdot 1_{n_1}], \lambda ^{-1}\cdot [\lambda \cdot 1_{n_2}]\,)= (1_{n_1},1_{n_2}) \end{aligned}$$

for each $\lambda \in \mu _{\mathrm {mcd}(n_1,n_2)}$. Hence $\ker \xi = {{\,\mathrm{im}\,}}\iota$ and the sequence is exact at the node having the triple product.

The last map is given by $\zeta (u_1,u_2)= \textstyle [\det _1(u_1)]^{1/n_1} \cdot [\det _2(u_2)]^{1/n_2}$, which for $(u_1,u_2)$ in the fibered group product satisfies, by definition,

$$\begin{aligned} 1=\delta _1(u_1) / \delta _2(u_2) = \{\textstyle [\det _1(u_1)]^{1/n_1} \cdot [\det _2(u_2)]^{1/n_2}\}^{n_1n_2}= [\zeta (u_1,u_2)]^{n_1n_2}. \end{aligned}$$

(well-definedness). To see that $\ker \zeta \subset {{\,\mathrm{im}\,}}\xi$, take $(u_1,u_2)$ in the fibered product group satisfying $\zeta (u_1,u_2)= \textstyle [\det _1(u_1)]^{1/n_1} \cdot [\det _2(u_2)]^{1/n_2} =1$. This means that

4.30 $$\begin{aligned} \textstyle \lambda _0 : =[\det _{n_1} (u_1)]^{1/n_1} \qquad \text {and} \qquad \lambda _0^{-1}=[\det _{n_2}(u_2)]^{1/n_2} \end{aligned}$$

are consistent. Due to Eq. (4.30), conveniently used, both matrices $\lambda _0^{-1}\cdot u_1$ and $\lambda _0 \cdot u_2$ are special unitary, and we also obtain $(u_1,u_2)=\xi (\lambda _0, \lambda _0^{-1}\cdot u_1, \lambda _0 \cdot u_2 )$.

Finally, on the other hand,

$$\begin{aligned} (\zeta \circ \xi )(\lambda , m_1,m_2)= \zeta (\lambda m_1,\lambda ^{-1}m_2) =\textstyle [\det _{n_1} (\lambda 1_{n_1})]^{1/n_1} [\det _{n_2} (\lambda 1_{n_2})]^{1/n_2}=1 \end{aligned}$$

so the inverted injection holds $\ker \zeta \supset {{\,\mathrm{im}\,}}\xi$ too. $\square$

Lemma 4.13 extracts the Lie group part of $\mathrm {U}(n_1) \times _{\det } \mathrm {U}(n_2 )$. (This group appears in the description of the unimodular gauge group in Lemma 4.12.) Its proof was inspired by one of Chamseddine–Connes–Marcolli, but is different from it due to the presence of the tensor product of algebras, whilst [14, Prop. 2.16] or [17, Prop. 1.185] focus on unitarities of semi-simple algebras, $A_1\oplus A_2\oplus \ldots \oplus A_k$.

In particular for the Standard Model [17, Prop. 1.199], the unimodular gauge group is the well-known $\{ \mathrm {U}(1) \times \mathrm {SU}(2) \times \mathrm {SU}(3) \}/ \mu _6$ Standard Model gauge group (cf. also [56, §6.2]). The embedding of the group $\mu _6$ of sixth roots of unit in the Lie group is given by $\lambda \mapsto (\lambda ,\lambda ^3,\lambda ^2)$, as pointed out in [57, §11.2.1]. Our embedding of the roots of unit appearing in the above Lemma is different, since the determinant for tensor products of algebras is governed by another rule: $\det (a_1\otimes a_2)= [\det _{n_1}(a_1) ]^{n_2} \times [\det _{n_2}(a_1)]^{n_1}$ for each $a_1\otimes a_2 \in A_1\otimes A_2$. On the physical side, the origin of the two roots of unit groups in the exact sequence

$$\begin{aligned} 1\rightarrow \mu _3 \rightarrow \mathrm {U}(1) \times \mathrm {SU}(2) \times \mathrm {SU}(3) \rightarrow \mathrm {SU}(A_F) \rightarrow \mu _{12} \rightarrow 1 \quad [17, Seq. 1.661] \end{aligned}$$

characterizing the unimodular gauge group for the algebra of the Standard Model $A_F={\mathbb {C}}\oplus \mathbb {H}\oplus M_3({\mathbb {C}})$ is quite different: on the one hand, the group^10^$\mu _3$ comes from $M_3({\mathbb {C}})$; and on the other $\mu _{12}$ does depend also on the number of generations and the representation of fermions.

By way of contrast, an important one conceptually, we stress that for $\mathrm {SU}(n)$-Yang–Mills(–Higgs) finite geometries where one has $A_1=M_N({\mathbb {C}})$ and $A_2 =M_n({\mathbb {C}})$ (so $n_1=N$ and $n_2=n$ above), *n* is the ‘color’ analogue, the two (special) unitary factors in Proposition 4.8 or the unimodular analogue above, have a different nature. The $\mathrm {PU}(N)$ [resp. $\mathrm {SU}(N)$] describes the symmetry of the base (and could be understood as the finite-dimensional analogue of diffeomorphisms of a manifold) and $\mathrm {PU}(n)$ [resp. $\mathrm {SU}(n)$] along the fibers.

## Yang–Mills–Higgs Theory with Finite-Dimensional Algebras

The Higgs field being considered at the same footing with the gauge bosons is one of the appealing characteristics that is offered by the gauge theory treatment with NCG. We now recompute the results of Sect. 4, revoking the restriction $D_F=0$. The aim is a formula informed by Weitzenböck’s. The Weitzenböck formula, $D_\omega ^2= \Delta ^{{\mathbb {S}}\otimes E}+{\mathcal {E}}$, includes the Higgs $\Phi$ and extends Lichnerowicz’s formula, to the product of the spinor bundle ${\mathbb {S}}$ with a vector bundle *E*. It is given in terms of an endomorphism ${\mathcal {E}}$ in $\Gamma (\mathrm {End}({\mathbb {S}} \otimes E))$:

5.1 $$\begin{aligned} {\mathcal {E}}= \frac{1}{4} R\otimes 1 + 1\otimes \Phi ^2 - \sum _{i,j} \frac{1}{2} \mathrm {i}\Gamma ^i \Gamma ^j \otimes {\mathbb {F}}_{ij} +\sum _{j} \mathrm {i}\gamma _M \Gamma ^j\otimes \mathrm {ad}(\nabla _j^{{\mathbb {S}}\otimes E}) \Phi , \end{aligned}$$

where $(\nabla ^{\mathbb {S}\otimes E})_j$ and ${\mathbb {F}}_{ij}$ are locally the connection on ${\mathbb {S}} \otimes E$ and the curvature on *E*, respectively. Further, $\gamma _M$ is the chirality element or $\gamma _5$ in physicists’ speak. (See e.g., [57, Prop. 8.6] for a proof.)

### The Higgs Matrix Field

We now turn off the fuzzy-gauge part of the spectral triple in order to compute the fluctuations along the finite geometry *F*. These fluctuations are namely generated by the second summand in the original (in the sense, ‘unfluctuated’). Dirac operator of the product spectral triple $D= D_{\mathrm {f}}\otimes 1_F + \gamma _{\mathrm {f}}\otimes D_F = D_{\mathrm {f}}\otimes 1_F + \gamma \otimes 1_{M_N({\mathbb {C}})} \otimes D_F$ where $D_F=D^*_F \in M_n({\mathbb {C}})_{\mathrm {s.a}}$ is the Dirac operator of the finite geometry *F*.

#### Proposition 5.1

The inner fluctuations of the Dirac operator along the finite geometry *F* are

5.2 $$\begin{aligned} (\omega _{F} + J\omega _{F} J^{-1})(\Psi )= (\gamma \otimes \phi ) (\Psi ) + \epsilon '' (\gamma \otimes 1_{M_N({\mathbb {C}})} \otimes 1_{M_n({\mathbb {C}})}) \Psi ( 1_V\otimes \phi ), \end{aligned}$$

for each $\Psi \in {\mathcal {H}}=V\otimes M_N({\mathbb {C}})\otimes {\mathcal {A}}_F$. These are parametrized by $\phi \in M_N({\mathbb {C}})\otimes \Omega ^1_{D_F}(M_n({\mathbb {C}}))$. Also $\phi ^*=\phi$ holds.

#### Proof

As before, one computes the corresponding Connes’ 1-forms ${{{\mathsf {a}}}[\gamma _{\mathrm {f}}\otimes D_F, {{\mathsf {c}}}]}$ in terms of ${{\mathsf {a}}}= 1_V\otimes W\otimes a$ and ${{\mathsf {c}}}= 1_V\otimes T \otimes c$, being $W,T\in M_N({\mathbb {C}})$ and $a,c\in M_n({\mathbb {C}})$. Namely,

$$\begin{aligned} \omega _{F}&= {{\mathsf {a}}}[\gamma _{\mathrm {f}}\otimes D_F, {{\mathsf {c}}}]\\&= {{\mathsf {a}}}[\gamma \otimes 1_{M_N({\mathbb {C}})}\otimes D_F, {{\mathsf {c}}}]\\&= (1_V\otimes W \otimes a) \big [ \gamma \otimes 1_{M_N({\mathbb {C}})} \otimes D_F, 1_V \otimes T \otimes c \big ] \\&= \gamma \otimes W T \otimes a [D_F,c ] \end{aligned}$$

We rename $\phi := X \otimes a [D_F,c ]$, since *W*, *T* are arbitrary and their product can be replaced by any matrix $X\in M_N({\mathbb {C}})$. Thus, $\omega _{F}=\gamma \otimes \phi \in \Omega ^1_{\gamma _{\mathrm {f}}\otimes {D_F}}({\mathcal {A}})= M_N({\mathbb {C}})\otimes \Omega ^1_{{D_F}}(M_n({\mathbb {C}}))$ as claimed. Since from the onset $\gamma$ is self-adjoint, so must be $\phi$, since $\omega _{F}^*=\omega _{F}$ is required. The remaining part of the fluctuations acting on $v\otimes Y\otimes m \in V \otimes M_N({\mathbb {C}})\otimes M_n({\mathbb {C}})$ are

5.3 $$\begin{aligned}&(J \omega _{F} J^{-1}) (v\otimes Y\otimes m)\nonumber \\&\quad = \big ( (C\otimes *_N\otimes *_n ) ( \gamma \otimes \phi ) (C^{-1}\otimes *_N\otimes *_n ) \big )(v\otimes Y\otimes m ) \nonumber \\&\quad = (C\otimes *_N \otimes *_n ) (\gamma C^{-1}v \otimes X Y^*\otimes a [D_F,c ] m^*) \nonumber \\&\quad = C \gamma C^{-1}v \otimes Y X^*\otimes (a [D_F,c ] m^* )^* \nonumber \\&\quad = \epsilon '' \gamma v \otimes Y X^*\otimes m (a [D_F,c ])^* , \end{aligned}$$

since $C \gamma = \epsilon '' \gamma C$ (cf. table of Def. 2.1). Therefore,

5.4 $$\begin{aligned} (J \omega _{F} J^{-1}) (\Psi )&= \epsilon ''\{\gamma \otimes 1_{M_N({\mathbb {C}})}\otimes 1_{M_n({\mathbb {C}})}\} (\Psi ) \{ (1_V\otimes X^* \otimes (a [D_F,c ] )^* \}\nonumber \\&= \epsilon '' \{\gamma \otimes 1_{M_N({\mathbb {C}})}\otimes 1_{M_n({\mathbb {C}})}\} (\Psi ) ( 1_V\otimes \phi ), \end{aligned}$$

since $\phi = X \otimes a [D_F,c ]$ is self-adjoint, as argued before. $\square$

In Eq. (5.3) of the proof, one could also have computed directly, using the explicit formula (3.4) for the chirality:

$$\begin{aligned} C\gamma C^{-1}&= (C \sigma (\eta ) \gamma ^0 C^{-1}) (C \gamma ^1 C^{-1})( C \gamma ^2 C^{-1})( C \gamma ^3 C^{-1}) \\&= (C {\sigma (\eta )} \gamma ^0 C^{-1}) \gamma ^1\gamma ^2\gamma ^3 = \overline{\sigma (\eta )} \gamma ^0\gamma ^1\gamma ^2\gamma ^3 = \pm \gamma . \end{aligned}$$

The complex conjugate in the last line appears since *C* is anti-linear. The sign is chosen as follows: notice that $\overline{\sigma (\eta )}$ is purely imaginary for the (1,3) and (3,1) signatures (and otherwise it is a sign). This means that the sign ± in last equation is $(-1)^{\#\text {number of minus signs in }\eta }=(-1)^q$. This different way to compute leads to the same result as the one given in the proof. Indeed, for four-dimensional geometries $(-1)^q$ is precisely $\epsilon ''$, according to the sign table in Definition 2.1, namely $\epsilon ''=-1$ for KO-dimensions 2 and 6 and $\epsilon ''=+1$ for KO-dimensions 0 and 4.

From Proposition 5.1 and Theorem 4.2, the form of the most general fluctuated Dirac operator follows: 5.5a5.5b5.5c5.5d5.5e We will call $\Phi \in M_N({\mathbb {C}})_\mathrm {s.a}\otimes \{\Omega ^1_{D_F}[M_n({\mathbb {C}})]\}_\mathrm {s.a}\subset \big \{ M_N[\Omega ^1_{D_F}(M_n({\mathbb {C}}))]\big \}_{\mathrm {s.a.}}$ the *Higgs field*, since in the smooth Riemannian case (where the analogous relation reads $\Phi ^{(C^\infty )}=D_F + J_F \phi ^{(C^\infty )} J_F^{-1}$) its analogue in the context of almost-commutative geometries leads to the Standard Model Higgs field, when the finite algebra ${\mathcal {A}}_F$ is correctly chosen (cf. [14, 57]).

#### Corollary 5.2

The fluctuated Dirac operator $D_{\omega }$ on the ‘flat’ () fuzzy space factor  of a gauge matrix geometry  satisfies5.6

#### Proof

$(D_\omega )^2= D_{{\mathrm{gauge}}}^2 + D_{\mathrm{Higgs}}^2 + \{D_{{\mathrm{gauge}}},D_{\mathrm{Higgs}}\}$. The gauge part $D_{{\mathrm{gauge}}}^2$ is known from Proposition 4.6; on the other hand, $D_{\mathrm{Higgs}}^2= (\gamma \otimes \Phi )^2=1_V\otimes \Phi ^2$ from the axiom in Definition 2.1 for the chirality $\gamma$. Finally, $\{D_{{\mathrm{gauge}}},D_{\mathrm{Higgs}}\}=$ , since $\gamma ^\mu \gamma = - \gamma \gamma ^\mu$. $\square$

Notice also that $\gamma ^{{{\hat{\mu }}}}=\gamma ^\alpha \gamma ^\rho \gamma ^\sigma$ anti-commutes with $\gamma$, for

$$\begin{aligned} \gamma ^\alpha \gamma ^\rho \gamma ^\sigma \gamma = - \gamma ^\alpha \gamma ^\rho \gamma \gamma ^\sigma = + \gamma ^\alpha \gamma \gamma ^\rho \gamma ^\sigma = - \gamma \gamma ^\alpha \gamma ^\rho \gamma ^\sigma . \end{aligned}$$

Since the matrices $\gamma ^\mu$ and $\gamma ^{{{\hat{\mu }}}}, \mu \in \Delta _4,$ span (the projection to *V* of) $D_{{\mathrm{gauge}}}$, the anti-commutator $\{D_{{\mathrm{gauge}}},D_{\mathrm{Higgs}}\}$ is traceless also if the fuzzy space is ‘curved,’ $X\ne 0$.

**Table 3:**
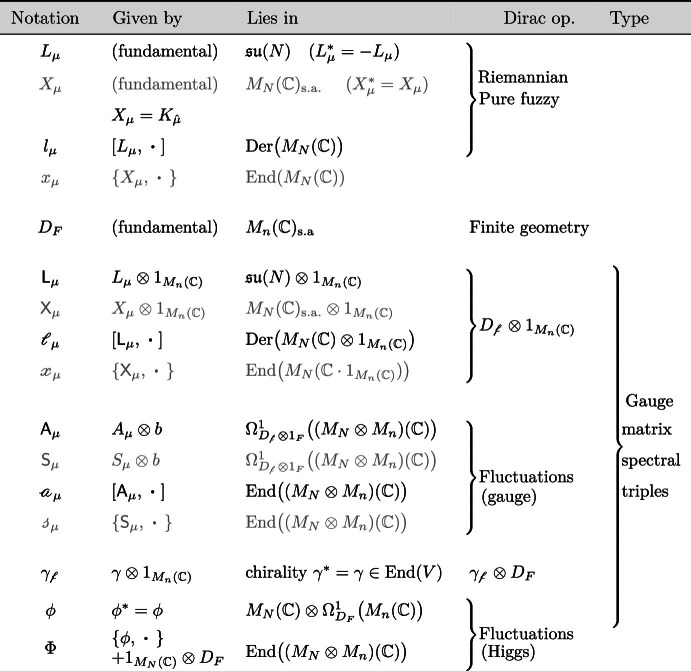
Notation for the matrices parametrizing the Dirac operator of *Riemannian* four-dimensional Yang–Mills–Higgs matrix spectral triples and its fluctuations along ${D=D_{\mathrm {f}}\otimes 1_{F} + \gamma _{\mathrm {f}}\otimes D_F}$, which are split into blocks along the gauge (${D_{\mathrm {f}}\otimes 1_{F}}$) and Higgs parts (${\gamma _{\mathrm {f}}\otimes D_F}$)

The accompanying gamma-matrices in the former case are omitted. The rows in gray will not be used below (*X* is set to zero, and this implies the vanishing of the rest of operators in gray rows). See Eq. (5.5) for more details

### Transformations of the Matrix Gauge and Higgs Fields

Throughout this section, we always assume the Riemannian signature. We now compute the effect of the gauge transformations, already explicitly known for the Dirac operator, on the field strength ${\mathscr {F}}_{\mu \nu }$ and on the Higgs field. For the former, this requires to know how the matrices ${\mathsf {A}}_\mu$ transform under ${\mathsf {G}}({\mathcal {A}};J)={\mathcal {U}}({\mathcal {A}})/ {\mathcal {U}}({\mathcal {A}}_J)$. We can pick a representing element of ${\mathsf {G}}({\mathcal {A}};J)$ in $u\in {\mathcal {U}}({\mathcal {A}})$ directly, since the apparent ambiguity up to an element $z\in {\mathcal {U}}({\mathcal {A}}_J)$ leads to^11^

$$\begin{aligned} \omega ^{uz}&=(uz)\omega (uz)^* + uz [D,(uz)^*] \\&= u \omega u^* + uz \big \{ [D,z^*]u^*+ z^*[D,u^*] \big \}\\&= u \omega u^* + uzz^*[D,u^*]= \omega ^{u}. \end{aligned}$$

The last line is obtained since ${\mathcal {U}}({\mathcal {A}}_J)= {\mathcal {U}}( {\mathcal {Z}}({\mathcal {A}}))$, so *z* is central (and thus $z^*$ too). Hence, $[D,z^*]=0$ (Table 3).

Next, observe that, by definition, and also by Jacobi identity on $M_{N\otimes n}({\mathbb {C}})$,5.7with analogous expressions for  and . This allows to write the field strength as the commutator with another quantity ${\mathsf {F}}_{\mu \nu } \in M_{N\otimes n}({\mathbb {C}})$ that we call *field strength matrix*, 5.8a5.8b

We now find the way the field strength transforms under the gauge group. By definition, the transformed field strength is given by the expression ${\mathscr {F}}_{\mu \nu }$ evaluated in the transformed potential , this latter being dictated by the way the Dirac operator transforms under ${\mathsf {G}}({\mathcal {A}};J)$. Specifically, 5.9a5.9b5.9c5.9dConcerning the Higgs, we come back to Eq. (5.5). We deduce from there and from (4.20), that the matrix field $\phi$, which parametrizes by (5.5e) the Higgs field, transforms like

5.9e $$\begin{aligned} \phi \mapsto \phi ^u = u \phi u^* + u[D_F,u^*]\, ,\qquad u\in {\mathsf {G}}({\mathcal {A}};J). \end{aligned}$$

The transformation of the field strength is more interesting:

#### Proposition 5.3

In Riemannian signature, the field strength of a Yang–Mills(–Higgs) finite geometry transforms under the gauge group as follows:5.10which is completely determined by the next transformation rule on the field strength matrix

5.11 $$\begin{aligned} {\mathsf {F}}_{\mu \nu } \mapsto {\mathsf {F}}^u_{\mu \nu }&= u {\mathsf {F}}_{\mu \nu } u^* = \mathrm {Ad}_u({\mathsf {F}}_{\mu \nu }) . \end{aligned}$$

#### Proof

Observe that for the pair  the same argument given about Eq. (5.7) for the pair  holds, and so does for the other pair of composition commutators appearing in the *u*-transformed field strength. Therefore, we can indeed write it in terms of the matrix ${\mathsf {F}}_{\mu \nu }^u:=[{\mathsf {L}}_\mu ,{\mathsf {L}}_\nu ] +[{\mathsf {L}}_\mu ,{\mathsf {A}}_\nu ^u ] - [\mathsf L_\nu ,{\mathsf {A}}^u_\mu ] + [{\mathsf {A}}_\mu ^u,{\mathsf {A}}_\nu ^u]$ as follows:5.12We now compute the transformed field strength matrix and all those terms that imply ${\mathsf {A}}$ (namely the transformation under *u* of ${\mathsf {T}}_{\mu \nu }:= {\mathsf {F}}_{\mu \nu } -[{\mathsf {L}}_\mu ,{\mathsf {L}}_\nu ] \rightarrow {\mathsf {T}}_{\mu \nu }^u$) and infer from that those the gauge transformations on the field strength matrix ${\mathsf {F}}_{\mu \nu }$.

$$\begin{aligned} {\mathsf {T}}^u_{\mu \nu }&= +\big [{\mathsf {L}}_\mu , \mathrm {Ad}_u({\mathsf {A}}_\nu ) + u[{\mathsf {L}}_\nu ,u^*] \big ] -\big [{\mathsf {L}}_\nu , \mathrm {Ad}_u({\mathsf {A}}_\mu ) + u[{\mathsf {L}}_\mu ,u^*] \big ]\\&\quad + \Big [ \mathrm {Ad}_u({\mathsf {A}}_\mu ) + u[{\mathsf {L}}_\mu ,u^*], \mathrm {Ad}_u({\mathsf {A}}_\nu ) + u[{\mathsf {L}}_\nu ,u^*] \Big ] \\&= +\big [{\mathsf {L}}_\mu , u {\mathsf {A}}_\nu u^* \big ]+ \big [{\mathsf {L}}_\mu , u [{\mathsf {L}}_\nu , u^*] \big ] - \big [{\mathsf {L}}_\nu , u {\mathsf {A}}_\mu u^* \big ] - \big [{\mathsf {L}}_\nu , u [{\mathsf {L}}_\mu ,u^*] \big ] \\&\quad + \big [ u{\mathsf {A}}_\mu u^*, u {\mathsf {A}}_\nu u^*\big ] + \big [ u [{\mathsf {L}}_\mu , u^*], u{\mathsf {A}}_\nu u^*\big ] \\&\quad + \big [ u[{\mathsf {L}}_\mu ,u^*], u[{\mathsf {L}}_\mu ,u^*]\big ] + \big [u{\mathsf {A}}_\mu u^*, u[{\mathsf {L}}_\nu ,u^*] \big ] \end{aligned}$$

The contributions to ${\mathsf {T}}^u_{\mu \nu }$ split into three: ${\mathsf {L}}{\mathsf {A}}$-terms (i.e., containing ${\mathsf {L}}_\mu ,{\mathsf {A}}_\nu$ or ${\mathsf {L}}_\nu ,{\mathsf {A}}_\mu$), ${\mathsf {A}}{\mathsf {A}}$-terms, and ${\mathsf {L}}{\mathsf {L}}$-terms. We compute them separately:

$$\bullet $$
$${\mathsf {A}}{\mathsf {A}}$$-terms: $$ \big [ u{\mathsf {A}}_\mu u^*, u {\mathsf {A}}_\nu u^*\big ]= u [{\mathsf {A}}_\mu ,{\mathsf {A}}_\nu ] u^*$$, clearly
$$\bullet $$
${\mathsf {L}}{\mathsf {L}}$-terms: When the commutators are expanded, the next ${\mathsf {L}}{\mathsf {L}}$-terms
  $$\begin{aligned} \big [{\mathsf {L}}_\mu , u [{\mathsf {L}}_\nu , u^*] \big ] - \big [{\mathsf {L}}_\nu , u [{\mathsf {L}}_\mu ,u^*] \big ] + \big [ u[{\mathsf {L}}_\mu ,u^*], u[{\mathsf {L}}_\mu ,u^*]\big ] \end{aligned}$$
  yield the quantity in bracelets, which can be neatly rewritten:
  $$\begin{aligned} \left\{ \begin{array}{ll} &{} {\mathsf {L}}_\mu u {\mathsf {L}}_\nu u^*-{\mathsf {L}}_\mu {\mathsf {L}}_\nu -u {\mathsf {L}}_\nu u^* {\mathsf {L}}_\mu + {\mathsf {L}}_\nu {\mathsf {L}}_\nu \\ &{}\quad +{\mathsf {L}}_\nu {\mathsf {L}}_\mu - {\mathsf {L}}_\nu u {\mathsf {L}}_\mu u^* +u{\mathsf {L}}_\mu u^* {\mathsf {L}}_\nu - {\mathsf {L}}_\mu {\mathsf {L}}_\nu \\ &{}\quad +u {\mathsf {L}}_\mu {\mathsf {L}}_\nu u^* -u {\mathsf {L}}_\nu {\mathsf {L}}_\mu u^* +{\mathsf {L}}_\mu {\mathsf {L}}_\nu -{\mathsf {L}}_\nu {\mathsf {L}}_\mu \\ &{}\quad +u {\mathsf {L}}_\nu u^* {\mathsf {L}}_\mu - {\mathsf {L}}_\mu u {\mathsf {L}}_\nu u^* + {\mathsf {L}}_\nu u {\mathsf {L}}_\mu u^* -u{\mathsf {L}}_\mu u^* {\mathsf {L}}_\nu \end{array} \right\} =u[{\mathsf {L}}_\mu ,{\mathsf {L}}_\nu ] u^*-[{\mathsf {L}}_\mu ,{\mathsf {L}}_\nu ] \end{aligned}$$
$$\bullet $$
${\mathsf {L}}{\mathsf {A}}$-terms: $\big [{\mathsf {L}}_\mu , u {\mathsf {A}}_\nu u^* \big ] \!-\! \big [{\mathsf {L}}_\nu , u {\mathsf {A}}_\mu u^* \big ]\!+\!\big [ u [{\mathsf {L}}_\mu , u^*], u{\mathsf {A}}_\nu u^*\big ] \!+\!\big [u{\mathsf {A}}_\mu u^*, u[{\mathsf {L}}_\nu ,u^*] \big ]$. This can be also obtained expanding the commutators as above; the last two commutators yield $u\{[{\mathsf {L}}_\mu ,{\mathsf {A}}_\nu ]-[{\mathsf {L}}_\nu ,{\mathsf {A}}_\mu ]\}u^* + r({\mathsf {L}},{\mathsf {A}})$. The excess terms $r({\mathsf {L}},{\mathsf {A}})$ are actually cancelled out with the two first propagators, yielding for the final expression of the ${\mathsf {L}}{\mathsf {A}}$-terms:
  5.13 $$\begin{aligned} u\big ([{\mathsf {L}}_\mu ,{\mathsf {A}}_\nu ]-[{\mathsf {L}}_\nu ,{\mathsf {A}}_\mu ]\big )u^* \end{aligned}$$

In view of the last equalities, we can conclude that

5.14 $$\begin{aligned} {\mathsf {T}}^u_{\mu \nu }&= u {\mathsf {T}}_{\mu \nu } u^* + u [{\mathsf {L}}_\mu ,{\mathsf {L}}_\nu ] u^*- [{\mathsf {L}}_\mu ,{\mathsf {L}}_\nu ]\\&= \mathrm {Ad}_u({\mathsf {T}}_{\mu \nu }) + \mathrm {Ad}_u \big ([{\mathsf {L}}_\mu ,{\mathsf {L}}_\nu ]\big )- [{\mathsf {L}}_\mu ,{\mathsf {L}}_\nu ] , \nonumber \end{aligned}$$

which, re-expressed in terms of ${\mathsf {F}}$, yields ${\mathsf {F}}_{\mu \nu }\rightarrow {\mathsf {F}}_{\mu \nu }^u = \mathrm {Ad}_u ({\mathsf {F}}_{\mu \nu })$. $\square$

#### Remark 5.4

Notice that ${\mathsf {L}}_\mu$ being the fuzzy analogue of the derivatives, the ‘surprising term’ $[{\mathsf {L}}_\mu ,{\mathsf {L}}_\nu ]$ is the analogue^12^ of $[\partial _\mu , \partial _\nu ]$, which is identically zero on the algebra $C^\infty (M)$. This seems to (but, as we will see, does not) imply the freedom of choice as to whether we take the field strength matrix as defined above by ${{\mathsf {F}}}_{\mu \nu }$, or rather $\tilde{{\mathsf {F}}}_{\mu \nu } = {{\mathsf {F}}}_{\mu \nu } -[{\mathsf {L}}_\mu , {\mathsf {L}}_\nu ]$ (called ${{\mathsf {T}}}_{\mu \nu }$ above). According to Eq. (5.14), $\tilde{{\mathsf {F}}}_{\mu \nu }$ transforms then as

5.15 $$\begin{aligned} \tilde{{\mathsf {F}}}_{\mu \nu } \mapsto \tilde{{\mathsf {F}}}^u_{\mu \nu }&= \mathrm {Ad}_u({\mathsf {F}}_{\mu \nu }) + \underbrace{ \mathrm {Ad}_u \big ([{\mathsf {L}}_\mu ,{\mathsf {L}}_\nu ]\big )- [{\mathsf {L}}_\mu ,{\mathsf {L}}_\nu ]}_{\text {traceless}}. \end{aligned}$$

Although for quadratic actions the last two terms add up to a traceless quantity, higher powers of the Dirac operator would mix the gauge sector with others. This confirms that the definitions in Eqs. (4.12) and (5.8b) are correct. For only then, the pure gauge sector (i.e., powers of ${\mathscr {F}}$) obtained from ${{\,\mathrm{Tr}\,}}_{\mathcal {H}}(D^{2m}_{\omega _{\mathrm {f}}})$ would be expressible (see [41], and for $m=2$, Eq. (2.10) above) as a sum over chord diagrams $\xi$, with $\varvec{\mu }= (\mu _1,\ldots ,\mu _m)$, $\varvec{\nu }= (\nu _1,\ldots , \nu _m)$,

5.16 $$\begin{aligned} \sum _{\begin{array}{c} \xi \\ \,\,m\text {-chord diag.} \end{array}}\sum _ {\varvec{\mu },\varvec{\nu }} \xi ^{\mu _1 \nu _1 \mu _2 \nu _2 \ldots \mu _m\nu _m} {{\,\mathrm{Tr}\,}}_{M_{N\otimes n}^{{\mathbb {C}}}}\{ {\mathscr {F}}^u_{\mu _1\nu _1} \cdots {\mathscr {F}}^u_{\mu _m\nu _m} \}. \end{aligned}$$

The scalars $\xi ^{\mu _1 \nu _1 \mu _2 \nu _2 \ldots \mu _m\nu _m}$ are expressed as sums of *m*-fold products of the bilinear form $\eta ^{\alpha \sigma }$ (signature) and are irrelevant for the discussion. The important conclusion is that, due to Proposition 5.3, the traced quantity is gauge invariant, since the transformation rule ignores the ‘space-time indices’ $\mu _i$ and $\nu _i$. The quartic computation is explicitly given below.

### Traces of Powers of *D*

The next statement is obvious:

#### Lemma 5.5

The fully fluctuated Dirac operator on the Yang–Mills–Higgs matrix spectral triple satisfies in ‘flat space’ (i.e., $X=0$),5.17

#### Proof

First, , is traceless by $\{\gamma ,\gamma ^\mu \}=0$. Now, from Eq. (5.6), since tracing the first summand yields, due to index symmetry of $\eta$ and index skew-symmetry of the field strength,

$$\begin{aligned} \sum _{\mu ,\nu }{{\,\mathrm{Tr}\,}}_{\mathcal {H}}(\gamma ^\mu \gamma ^\nu \otimes {\mathscr {F}}_{\mu \nu } )=\sum _{\mu ,\nu } \eta ^{\mu \nu }\dim V {{\,\mathrm{Tr}\,}}_{M_{N\otimes n}^{{\mathbb {C}}}}{\mathscr {F}}_{\mu \nu } = 0 \end{aligned}$$

one gets the result by Eq. (4.14). $\square$

#### Lemma 5.6

The fully fluctuated Dirac operator of the Yang–Mills–Higgs finite geometry satisfies in ‘flat space’ (i.e., $X=0$),5.18

#### Proof

Squaring the expression for $(D_\omega |_{X=0})^2$ given by Lemma 5.2.for some sign ± in last line, which is in fact irrelevant since ${{\,\mathrm{Tr}\,}}_{V}( \gamma ^\mu \gamma ^\nu \gamma ^\rho \gamma )=0$ for any choice of indices. The line before the last is also traceless. Further, using ${{\,\mathrm{Tr}\,}}_{V}(\gamma ^\mu \gamma ^\nu \gamma ^\alpha \gamma ^\rho )$ given in Eq. (2.10),5.19By symmetry of $\eta$ and skew-symmetry of ${\mathscr {F}}$, the first chord diagram vanishes, and by the same token, also the second line in Eq. (5.19). The second chord diagram comes with a minus sign and, using the skew-symmetry ${\mathscr {F}}$, one can see that the third diagram yields the same contribution, namely $\sum _{\mu ,\nu ,\rho ,\sigma }(-\eta ^{\nu \rho }\eta ^{\mu \alpha }) {{\,\mathrm{Tr}\,}}_{M_{N\otimes n}^{{\mathbb {C}}}}({\mathscr {F}}_{\mu \nu }{\mathscr {F}}_{\alpha \rho })$. For the second to last line, ${{\,\mathrm{Tr}\,}}_{V}(\gamma ^\mu \gamma \gamma ^\nu \gamma )=-\eta ^{\mu \nu }{{\,\mathrm{Tr}\,}}_{V}1_V$. Dividing the whole Eq. (5.19) by $\dim V=4$ and get the claim. $\square$

## The Spectral Action for Yang–Mills–Higgs Matrix Spectral Triples: Toward the Continuum Limit

We now give the main statement and, after its proof, we compare it with [13, §2], which derives from NCG the Yang–Mills–Higgs theory over a smooth manifold. Since in differential geometry the Einstein summation convention is common, we restore it here (also in the fuzzy context) together with the raising and lowering of indices with the constant signature $\eta ^{\mu \nu }=(\eta _{\mu \nu }^{-1})$ and $\eta _{\mu \nu }$. Using the lemmata of previous sections, we can give a short proof to the main result:

### Theorem 6.1

For a Yang-Mills–Higgs matrix spectral tripleon a 4-dimensional flat ($X=0$) Riemannian ($p=0$) fuzzy base, the Spectral Action for a real polynomial $g(x)= \frac{1}{2} \sum _{i=1}^m a_i x^i$ reads

6.1 $$\begin{aligned} \frac{1}{4}{{\,\mathrm{Tr}\,}}_{\mathcal {H}}g(D) = S^\mathrm {f}_{\textsc {ym}}+ S^\mathrm {f}_{\mathrm {H}}+ S^\mathrm {f}_{\mathrm {g\text {-}H}}+ S^\mathrm {f}_\vartheta + \ldots , \end{aligned}$$

where each sector is defined as follows:6.2and the rest terms in the ellipsis represents operators  being  of order $\ge 5$. Further, $g_{\mathrm {e}}$ is the even part of the polynomial *g* truncated to degree $<5$.Moreover, one obtains positivity for each of the following functionals, independently:

$$\begin{aligned} S^\mathrm {f}_{\vartheta },\, S^\mathrm {f}_{\textsc {ym}}, \, S^\mathrm {f}_{\mathrm {H}}\ge 0\,, \qquad \text {if } a_4\ge 0 \,. \end{aligned}$$

### Proof

Recall $D=D_{{\mathrm{gauge}}}+D_{\mathrm{Higgs}}$. It is obvious that ${{\,\mathrm{Tr}\,}}_{\mathcal {H}}(D)=0$. The possible crossed-products contributions to ${{\,\mathrm{Tr}\,}}(D^3)$ are ${{\,\mathrm{Tr}\,}}(D_{{\mathrm{gauge}}}^2D_{\mathrm{Higgs}})$ and ${{\,\mathrm{Tr}\,}}(D_{{\mathrm{gauge}}} D_{\mathrm{Higgs}}^2)$. The former vanishes because in spinor space *V* we have to trace over $\gamma ^\mu \gamma ^\nu \gamma$, which vanishes. Similarly, $D_{\mathrm{Higgs}}^3$ is traceless since $\gamma ^3=\gamma$ is, and and $D_{{\mathrm{gauge}}}^3$ vanishes by ${{\,\mathrm{Tr}\,}}_{V}(\gamma ^\mu \gamma ^\nu \gamma ^\rho )=0$. Thus odd powers of *D* are traceless, at least for degrees $<5$.

Hence inside the trace over ${\mathcal {H}}$, *f* can be replaced by its even part $f_{\mathrm {e}}$. Notice that by Lemmas 5.5 and 5.6, thenObserve that by definition, Eq. (4.14), , so the last term yields by expansion of the commutators and cyclicity,6.3The result follows by inserting the definitions from Eq. (6.2) and by observing that the trace of $D^6$ is a noncommutative polynomial (which we do not determine) of homogeneous degree 6 in the eight letters  and ; this is, in the worse case, the rest term in (6.1).

Regarding positivity: First, notice that  is a positive operator, and that so is $\vartheta \in \mathrm {End}( M_N({\mathbb {C}})\otimes {\mathcal {H}}_F)$ by the same token, . Thus $f_{\mathrm {e}}(\sqrt{\vartheta })$ is well-defined and its trace positive, since $f_{\mathrm {e}}$ is by definition an even polynomial.

Further relations like , and similar ones for all the commutators defining the field strength, lead to ${\mathscr {F}}_{\mu \nu }^* = -e_\mu e_\nu {\mathscr {F}}_{\mu \nu }$. Since $\eta =\mathrm {diag}(e_0,\ldots , e_3)$, one obtains the positivity of the operator

6.4 $$\begin{aligned} -{\mathscr {F}}_{\mu \nu } {\mathscr {F}}^{\mu \nu } = {\mathscr {F}}_{\mu \nu } (-e_\mu e_\nu {\mathscr {F}}_{\mu \nu } ) ={\mathscr {F}}_{\mu \nu }({\mathscr {F}}_{\mu \nu })^* \ge 0,\,\qquad \text {(no sum)}. \end{aligned}$$

Therefore, also the positivity holds summing over $\mu ,\nu$, which is a positive multiple of ${S^\mathrm {f}_{\textsc {ym}}}$, whose positivity also follows. Similarly, since $\Phi$ is self-adjoint, even powers of it are positive, thus so is ${S^\mathrm {f}_{\mathrm {H}}}$. $\square$

We now comment on the interpretation of this result. For fuzzy geometries, the equivalent of integration over the manifold is tracing operators $M_N({\mathbb {C}})\rightarrow M_N({\mathbb {C}})$. (At the risk of being redundant, notice that the unit matrix in that space has trace $N^2$.) First, recall that $\Phi$ is self-adjoint. We identify the Higgs field *H* on a smooth, closed manifold *M* with $\Phi$, so the quartic part $\int _M |H|^4 \mathrm {vol}$ of the potential for the Higgs is ${{\,\mathrm{Tr}\,}}_{M_{N\otimes n}^{{\mathbb {C}}}}( \Phi ^4)$. In the Riemannian case, in order to address the gauge-Higgs sector^13^, notice that since $\Phi =\Phi ^*$, if $a_4=1$,6.5This interpretation of  as the covariant derivative ${\mathbb {D}}_\mu =\partial _\mu + {\mathbb {A}}_\mu$ for Yang–Mills connection, with the local gauge potential ${\mathbb {A}} _\mu$ absorbing the coupling constant (cf. Def. 4.5 and Remark 5.4).

Next, notice that ${\mathscr {F}}_{\mu \nu }$ is a matrix-version of the $\mathrm {SU}(n)$-Yang–Mills (local) curvature ${\mathbb {F}}_{\mu \nu }$ for the action $S_{\textsc {ym}}$. If $a_4=1$, one has the exact correspondence 6.6a6.6b For the time being, the previous identifications hold only the Riemannian signature, since for $(p,q)\ne (0,4)$ anti-commutators appear; these, unlike commutators, are no longer derivations in the algebraic sense. Nevertheless, keeping this caveat in mind, we extend the previously defined functionals to any signature (there, each  is replaced by ). It holds then in general signature.

**Table 4:** Only in this table, ${{\,\mathrm{Tr}\,}}{\mathscr {P}}$ denotes the trace of operators ${\mathscr {P}}:M_{N\otimes n}({\mathbb {C}})\rightarrow M_{N\otimes n}({\mathbb {C}})$; *gauge potential* means the local expression for the connection

Meaning	Random matrix case (Riemannian signature)	Smooth operator
Derivation	$\ell _{\mu }$	$\partial _{i}$
Gauge potential		$\mathbb {A}_{i}$
Higgs field	$\Phi$	*H*
Covariant Derivative		$\mathbb {D}_{i}=\partial _{i}+\mathbb {A}_{i}$
Field strength		$[\mathbb {D}_{i}, \mathbb {D}_{j}]=\mathop {\overbrace{\partial _{i}, \partial _{j}}}\limits ^{\equiv 0}+ \partial _{i}\mathbb {A}_{j}-\partial _{j}\mathbb {A}_{i}+[\mathbb {A}_{i}, \mathbb {A}_{j}]$
Higgs lagrangian	$\hbox {Tr}(a_{2}\Phi ^{2}+a_{4}\Phi ^{4})$	$\int _{M}(a_{2}|H|^{2}+a_{4}|H|^{4})\hbox {vol}$
Gauge-Higgs coupling		$\int _{M}|\mathbb {D}_{i}H|^{2}\hbox {vol}$
Yang–Mills action	$-\frac{1}{4}\hbox {Tr}\mathscr {F}_{\mu \nu }\mathscr {F}^{\mu \nu }$	$-\frac{1}{4}\int _{M}\hbox {Tr}_{\mathfrak {su}(n)}(\mathbb {F}_{ij}\mathbb {F}^{ij})\hbox {vol}$

Finally, $$a_2 $$ and $$a_4$$ stand for for real parameters in *f* in Theorem 6.1 which are particularly relevant for the Higgs Lagrangian, see Eq. (6.2). The analogies implying  hold only for the Riemannian signature

The identification of  in the Riemannian case (and its extension  to the general signature) with the curvature ${\mathbb {F}}_{\mu \nu }=\partial _\mu {\mathbb {A}}_\nu -\partial _\nu \mathbb A_\mu + [{\mathbb {A}}_\mu ,{\mathbb {A}}_\nu ]$ of the smooth case is further supported by the fact that  generalizes the multiplication operator $\partial _\mu {\mathbb {A}}_\nu$, on top of the reason already given in Remark 5.4. The alternative to this definition, using only  in place of  (and similar replacements), yields instead $(\partial _{\mu } \circ {\mathbb {A}}_{\nu }) \psi = (\partial _\mu {\mathbb {A}}_\nu ) \cdot \psi +{\mathbb {A}}_\nu \partial _\mu (\psi )$ on sections $\psi$ (fermions). Notice also that for the smooth field strength one gets the positivity of the type of Eq. (6.4), namely $-{{\,\mathrm{Tr}\,}}_{\mathfrak {su}(n)} ({\mathbb {F}}_{\mu \nu } \overline{{\mathbb {F}}^{\mu \nu }}) \ge 0$, due to $\mathbb {F}_{\mu \nu } \overline{\mathbb {F}^{\mu \nu }}=-\mathbb {F}_{\mu \nu }\mathbb {F}_{\mu \nu }$ [17, below Eq. 1.597]. We summarize this section in Table 4.

### Remark 6.2

Notice that in the expression for the Yang–Mills action, when the model is fully expanded in terms of the fields  and , the next tetrahedral action appears6.7as well as the same type of action, , in the variable . The reference to a tetrahedron is justified when one writes that action in full,where the faint (blue) lines correspond to contractions of Greek indices and black lines to matrix-indices *i*, *j*, *m*, *l*. Modulo the restriction $\mu \ne \nu$ present in the sum, this kind of action  is an example of the ‘matrix-tensor model’ class [4].

## Conclusions

We introduced gauge matrix spectral triples, computed their spectral action and interpreted it as Yang–Mills–Higgs theory, if the inner-space Dirac operator is non-trivial (and as Yang–Mills theory if it is trivial), for the four-dimensional geometry of Riemannian signature. We justified this terminology based on Remark 5.4 and Sect. 6; in particular see Table 4 for the summary. The partition function of the Yang–Mills–Higgs theory is an integral over gauge potentials ${\mathsf {A}}_\mu$ and a Higgs field $\Phi$ in (subspaces of the) following matrix spaces

$$\begin{aligned} {\mathsf {A}}_\mu \in M_n(\Omega ^1_{\mathrm {f}}) \quad \text {and} \quad \phi \in M_N ( \Omega ^1 _F) \end{aligned}$$

where ${\Omega ^1_{\mathrm {f}}}$ and $\Omega ^1_F$ are the Connes’ 1-forms along the fuzzy and the finite geometry, respectively, both parametrized by (finite) matrices, see Sect. 8. Additionally, the partition function for the spectral action implies an integration over four copies of $\mathfrak {su}(N)$; each of these matrix variables ${\mathsf {L}}_\mu$ appears as the adjoint . These operators  are interpreted as degrees of freedom solely of the fuzzy geometry, in concordance with the identification of $\mathrm {Der}(M_N({\mathbb {C}}))$ with a finite version of the derivations on $C^\infty (M)$, that is, vector fields.

As in the almost-commutative setting $M \times F$, with *M* a smooth manifold, the Higgs field arises from fluctuations along the finite geometry *F* and the Yang–Mills gauge fields from those along the smooth manifold *M*. This is apparent in the parametrizing matrix subspaces (see Eq. (8.3)) for the matrix Higgs field and the matrix gauge potentials, which are swapped if one simultaneously^14^ exchanges $n \leftrightarrow N$ and ${F \leftrightarrow \mathrm {f}}$. The Yang–Mills–Higgs matrix theory has a projective gauge group ${\mathsf {G}}= \mathrm {PU}(N) \times \mathrm {PU}(n)$. The left factor corresponds with the symmetries of the fuzzy spacetime and the right one with those of the ‘inner space’ of the gauge theory (a similar interpretation holds for the unimodular gauge groups in Lemmas 4.12 and 4.13), so the whole group ${\mathsf {G}}$ could be understood as $C^\infty (M,\mathrm {SU}(n))$ after a truncation has been imposed on *M*. A rigorous interpretation, e.g., in terms of spectral truncations [20], is still needed.

Another approach to reach a continuum limit resembling smooth spin manifolds is the Functional Renormalization Group, which could be helpful in searching the fixed points (cf. the companion paper [42] for the application of this idea to general multimatrix models).

## Outlook

Aiming at a model with room for gravitational degrees of freedom, the careful construction of a Matrix Spin Geometry needs a separate study (in particular requiring $X_\mu \ne 0$ and thus also a more general treatment than that of Sect. 5). If that is concluded, one could identity for signature (0, 4)

$$\bullet $$
Lemma 3.3 with ‘Fuzzy Lichnerowicz formula,’
$$\bullet $$
Proposition 4.6 with ‘Fuzzy flat Weitzenböck formula,’ and
$$\bullet $$
Proposition 4.7 with ‘Fuzzy Weitzenböck formula’.

In order to give a more structured appearance to the partition function for Riemannian ($p=0$), flat Yang–Mills–Higgs spectral triples, we recall the dependence of our functionals on the *fundamental matrix fields* ${\mathsf {L}}_\mu ,{\mathsf {A}}_\mu$ and $\phi$. The ${\mathsf {L}}$’s are functioning as derivatives ${\mathsf {L}}_\mu \in \mathfrak {su}(N)\otimes 1_n$, and  is the derivation defined by the adjoint action, , for each $\mu$. One arrives at a similar situation with the *matrix gauge potentials*

$$\begin{aligned} {{\mathsf {A}}_\mu \in \big \{\Omega ^1_{D_{\mathrm {f}}}[ M_N({\mathbb {C}})]\big \}_{\mathrm {anti\text {-}Herm.}} \otimes M_n({\mathbb {C}})_{\mathrm {s.a.}} \subset M_N({\mathbb {C}})\otimes M_n({\mathbb {C}}),} \end{aligned}$$

where the subindex in the curly brackets restricts to anti-Hermitian 1-forms. In terms of these  is defined, again, via derivations: , which already bear a non-trivial factor in the inner space.^15^ This yields dependences . Further, by Eq. (5.5e), also $\Phi =\Phi (\phi )$. All in all, this yields for each sector

$$\begin{aligned} S^\mathrm {f}_{\textsc {ym}}&=S^\mathrm {f}_{\textsc {ym}}({\mathsf {L}},{\mathsf {A}}), \quad S^\mathrm {f}_{\mathrm {g\text {-}H}}=S^\mathrm {f}_{\mathrm {g\text {-}H}}({\mathsf {L}}, {\mathsf {A}}, \phi ), \\ S^\mathrm {f}_{\mathrm {H}}&= S^\mathrm {f}_{\mathrm {H}}(\phi ), \quad S^\mathrm {f}_\vartheta = S^\mathrm {f}_\vartheta ( {\mathsf {L}},{\mathsf {A}}). \end{aligned}$$

The partition function, using a polynomial *g*(*x*) , reads8.1where

$$\bullet $$
the Spectral Action is given by Theorem 6.1
$$\bullet $$
the partition function ${{\mathcal {Z}}^\mathrm {f}={\mathcal {Z}}_{N, n}^{\mathrm {f},f }}$ implies integration over the matrix space ${\mathscr {N}}$ that depends on the parameters *N* and *n* via
  8.2 $$\begin{aligned} ({\mathsf {L}}_\mu ,{\mathsf {A}}_\mu ,\phi ) \in {\mathscr {N}} = {\mathscr {N}}_{N,n}^{p=0,q=4} =\big [ \mathfrak {su}(N)\big ]^{\times 4}\times \big [{\mathscr {N}} ^{\mathrm {gauge}}_{N,n}\big ]^{\times 4} \times {\mathscr {N}} ^{\mathrm {Higgs}}_{ N,n } , \end{aligned}$$
  with the Higgs and gauge fields matrix spaces defined by 8.3a8.3b
$$\bullet $$
the measure $\mathrm {d}D=\mathrm {d}\mathsf {L}\, \mathrm {d}\mathsf {A} \,\mathrm {d}\phi$ is the product of Lebesgue measures on the three factors of (8.2).

While writing down the path integral does not solve the general problem of how to quantize noncommutative geometries, this finite-dimensional setting might pave one of the possible ways there, for instance, also by addressing these via computer simulations (Barrett-Glaser’s aim). However, it should be stressed that the treatment of this path integral is not yet complete, due to the gauge redundancy to be still taken care of. A suitable approach is the BV-formalism^16^ (after Batalin and Vilkovisky [12]), all the more considering that it has been explored for $\mathrm {U}(2)$-matrix models in [33], and lately also given in a spectral triple description [32].

En passant, notice that since the main algebra here is $$ M_N(A)$$ with *A* a noncommutative algebra, the Dyson–Schwinger equations of these multimatrix models would be ‘quantum’ (in the sense of Mingo-Speicher [38, §4]; this is work in progress).

## Appendix A. Proofs of Some Lemmata

### Proof of Lemma 3.2

For the first equation: If $\mu =\nu$, then

$$\begin{aligned} \gamma ^\mu \gamma ^{{{\hat{\mu }}}}=\gamma ^\mu \gamma ^0\cdots \widehat{\gamma ^\mu } \cdots \gamma ^3=(-1)^{\mu }\gamma ^0\cdots \gamma ^\mu \cdots \gamma ^3, \end{aligned}$$

since the first $\gamma ^\mu$ has to ‘jump’ $\mu$ gamma-matrices in order to form $\gamma ^0\gamma ^1\gamma ^2\gamma ^3$. If $\mu \ne \nu$, precisely the two gamma matrices with indices different from $\mu$ and $\nu$ survive, which explains $\delta _{\mu \nu \alpha \sigma }$. The LHS of Eq. (3.3a) contains also $(\gamma ^\mu )^2=e_\mu 1_V$. To justify the sign $(-1)^\mu \mathrm {sgn}(\nu -\mu )$, notice, by explicit computation, that if $\mu < \nu$ then the $(\mu ,\nu )$-pairs $(0,\nu )$ and $(2,\nu )$ yield positive sign, whereas $(1,\nu )$ negative. Thus the sign is $(-1)^{\mu }$ (in no case the sign depends on $\nu$, as far as $\mu < \nu$). The situation is inverted if $\mu > \nu$, where the pairs $(1,\nu )$ and $(3,\nu )$ yield positive sign and $(2,\nu )$ negative. Thus, the sign is $(-1)^{\mu -1}$.

Notice that in $\gamma ^{{{\hat{\mu }}}}\gamma ^\mu$ the last matrix has to move $4-\mu -1$ places to the right to arrive at the $\mu$-th factor, which says that $\gamma ^{{{\hat{\mu }}}}\gamma ^\mu =(-1)^{3-\mu } \gamma ^0 \gamma ^1\gamma ^2\gamma ^3=-(-1)^{\mu } \gamma ^0 \gamma ^1\gamma ^2\gamma ^3$ and, by Eqs. (3.3a), (3.3b) follows.

For Eq. (3.3c), one notices that $\gamma ^\mu$ has to jump, in order to pass to the other side, three matrices (one of which is $\gamma ^\mu$ itself), which yields the sign $(-1)^2$.

Regarding Eq. (3.3d) To obtain the second summand, notice that if $\mu =\nu$, then the LHS is of the form $( \gamma ^\iota \gamma ^\lambda \gamma ^\tau )^2$ with pairwise different indices (i.e., anti-commuting gamma-matrices). Therefore, $\gamma ^{{{\hat{\mu }}} }\gamma ^{{{\hat{\mu }}} } =e_\iota \gamma ^\lambda \gamma ^\tau \gamma ^\lambda \gamma ^\tau =-e_\iota \gamma ^\lambda \gamma ^\lambda \gamma ^\tau \gamma ^\tau = - e_\iota e_\lambda e_\tau 1_V$. Since each  is a sign and $\{\iota ,\lambda ,\tau ,\mu \}= \Delta _4$, by multiplying the last expression by $e_\mu ^2=1$ one arrives to $\gamma ^{{{\hat{\mu }}} }\gamma ^{{{\hat{\mu }}} }=-e_\mu \cdot (e_0e_1 e_2 e_3 ) 1_V = -e_\mu \det (\eta )1_V$.

If $\nu \ne \mu$, we first determine the corresponding RHS term up to a sign, and thereafter correct it. First, it is clear that $\gamma ^{{{\hat{\mu }}} }\gamma ^{{{\hat{\nu }}} }$ is a product of $\gamma ^\mu$ (which appears in $\gamma ^{{{\hat{\nu }}} }$), with $\gamma ^\nu$ (which appears in $\gamma ^{{{\hat{\nu }}} }$) and, additionally, with the other two gamma-matrices whose indices $\{\lambda ,\rho \}$ that are neither $\mu$ nor $\nu$. But each one of the latter appears twice, once in $\gamma ^{{{\hat{\nu }}} }$ and once in $\gamma ^{{{\hat{\mu }}} }$. The matrix is then proportional to $\gamma ^\mu \gamma ^\nu$, which with the squared matrices $\gamma ^\lambda$ and $\gamma ^\rho$ yield $\varsigma _{\mu \nu } e_\lambda e_\rho \gamma ^\mu \gamma ^\nu$ for $\lambda , \rho \in \Delta _4\setminus \{\mu ,\nu \}$ and $\lambda \ne \rho$, for a sign $\varsigma _{\mu \nu }=\pm$ that we now determine. To enforce the inequality of all the indices, we introduce $\delta _{\mu \nu \lambda \rho }$, but since $e_\lambda e_\rho \delta _{\mu \nu \lambda \rho }$ is symmetric in $\lambda$ and $\rho$, we have to divide the sum over those indices by 1/2. To find the correct sign $\varsigma _{\mu \nu }$, by explicit computation one sees that $\varsigma _{\mu \nu }=-1$ if and only if $(\mu ,\nu )$ is (0, 2), (2, 0), (3, 1) or (1, 3) and $\varsigma _{\mu \nu }=+1$ in all the other cases. That is, $\varsigma _{\mu \nu }=-1$ if and only if $|\mu -\nu |$ is even. But this is precisely equivalent to $\varsigma _{\mu \nu }=(-1)^{|\mu -\nu |+1}$. $\square$

### Lemma A.1

Let $A_i$ be unital, associative algebras, and let $Z(A_i)$ be the center of $A_i$. Then $Z(A_1\otimes A_2) = Z(A_1) \otimes Z( A_2)$.

### Proof

Notice that for (so far, arbitrary) $a_i, b_i \in A_i$ ($i=1,2$), one has by adding and subtracting $a_1b_1\otimes b_2a_2$ and rearranging,

A.1 $$\begin{aligned} {[}a_1 \otimes a_2 , b_1\otimes b_2 ] = [a_1,b_1] \otimes (b_2 a_2 ) + (a_1 b_1) \otimes [a_2,b_2]. \end{aligned}$$

Clearly, if $a_i\in Z(A_i)$ for $i=1,2$, then the RHS vanishes for each $b_i \in A_i$, that is, $a_1\otimes a_2\in Z ( A_1 \otimes A_2)$. Therefore $Z(A_1)\otimes Z(A_2) \subset Z ( A_1 \otimes A_2)$.

Conversely, notice that if $a_1\otimes a_2=0$, then we are done, so we suppose $a_1\otimes a_2 \in Z(A_1\otimes A_2)\setminus \{0\}$. If the LHS of the previous equation vanishes for each $b_1\otimes b_2 \in A_1\otimes A_2$, so does for $b_1=1$; in which case, one gets $a_1 \otimes [a_2,b_2]=0$ for each $b_2 \in A_2$, so $a_2\in Z(A_2)$, since $a_1\ne 0$ by assumption. Repeating the argument now taking $b_2=1$ instead, one gets $Z(A_1)\otimes Z(A_2) \supset Z ( A_1 \otimes A_2)$. $\square$

### Proof of Lemma 5.6

Again, in the whole proof we set $K=0$, even though the notation will not reflect it. This can be obtained by small modifications from the previous lemma: if $K_\mu =0$ for each $\mu$, then

$$\begin{aligned} \omega _{\mathrm {f}}(\Psi )&= {{\mathsf {a}}}[D_{\mathrm {f}}\otimes 1_F, {{\mathsf {a}}}' ] (\Psi ) = \sum _\mu \gamma ^{{{\hat{\mu }}}} v \otimes W [ X_\mu , T ] Y \otimes a c \psi \\&= \sum _\mu \big ( \gamma ^{{{\hat{\mu }}}}\otimes W [X_\mu , T] \otimes a c \big ) \Psi \\&= \sum _\mu \big ( \gamma ^{{{\hat{\mu }}}} \otimes S_\mu \otimes a c \big ) \Psi \end{aligned}$$

and $S_\mu :=W [X_\mu ,T]$. Since *a* and *c* are arbitrary matrices, we rename $b=a c$. Again, since the

A.2 $$\begin{aligned} (\gamma ^{{{\hat{\mu }}}} \otimes S_\mu \otimes b)^*&= e_{{{\hat{\mu }}}} \gamma ^{{{\hat{\mu }}}} \otimes S_\mu ^* \otimes b^* \end{aligned}$$

and since we had set already $b\in \mathrm {i}\,{\mathfrak {u}}(n)$ we conclude that $S_\mu ^* = e_{{{\hat{\mu }}}} S_\mu$. This sign is $e_{{{\hat{\mu }}}}=(-1)^{q+1}e_\mu$, according to [41, App. A]. It follows from the definition, that the anti-linear operator $C:V \rightarrow V$ satisfies $C(\gamma ^\alpha \gamma ^\rho \gamma ^\sigma ) C ^{-1}= C \gamma ^\alpha C^{-1}\cdot C \gamma ^\rho C^{-1}\cdot C \gamma ^\sigma C^{-1}=\gamma ^\alpha \gamma ^\rho \gamma ^\sigma$ for each triple of indices $\alpha ,\rho ,\sigma \in \Delta _4$. Therefore, $C\gamma ^{{{\hat{\mu }}}} C^{-1}= \gamma ^{{{\hat{\mu }}}}$ and the operator ${J\omega _{\mathrm {f}}J^{-1}}$ can thus readily be computed: for $\Psi =v\otimes Y\otimes \psi \in V \otimes M_N({\mathbb {C}})\otimes M_n({\mathbb {C}})$,

$$\begin{aligned} (J \omega _{\mathrm {f}}J^{-1}) (\Psi )&= \big ( J {{\mathsf {a}}}[D_{\mathrm {f}}\otimes 1_F, {{\mathsf {a}}}' ] J^{-1}\big ) (\Psi ) \\&= \sum _\mu (C\otimes *_N \otimes *_n ) (\gamma ^{{{\hat{\mu }}}} C^{-1}v \otimes S_\mu Y^* \otimes b \psi ^*)\\&= \sum _\mu (\underbrace{C \gamma ^{{{\hat{\mu }}}} C^{-1}}_{\gamma ^{{{\hat{\mu }}}}} v) \otimes (S_\mu Y^*)^* \otimes (b \psi ^*)^* \\&=\sum _\mu (\gamma ^{{{\hat{\mu }}}} \otimes 1_{M_N({\mathbb {C}})}\otimes 1_{M_n({\mathbb {C}})}) \Psi (1_V\otimes e_{{{\hat{\mu }}}} S_\mu \otimes b) \\&= \sum _\mu ( (-1)^{q+1}e_\mu \gamma ^{{{\hat{\mu }}}} \otimes 1_{M_N({\mathbb {C}})}\otimes 1_{M_n({\mathbb {C}})}) \Psi (1_V\otimes S_\mu \otimes b) . \end{aligned}$$

$$\square $$

## Footnotes

1 Also, other proposals related to discretizations or truncations [11, 25, 30, 31] are (closer to) spectral triples.

2 Here the name ‘base’ for is taken from the principal bundle $G \hookrightarrow P \rightarrow M$ terminology, where *M* is usually the spacetime manifold.

3 This is usually referred to as ‘finite spectral triple’ but in this paper all spectral triples are finite dimensional.

4 This is not the first time that the Connes–Chamseddine regulating function *f* does not appear and instead a polynomial is used, e.g., see the approach by [39] in the spin network context.

5 This condition does not appear in list given by Barrett and in fact follows from the construction of the explicit $\gamma$ matrices, so it is tautological but useful to emphasize, as it also appears in the smooth case [57]. Barrett also allows algebras ${\mathcal {A}}$ over ${\mathbb {R}},{\mathbb {C}}$ and ${\mathbb {H}}$; for quaternion coefficients, $M_{N/2}({\mathbb {H}})\subset M_{N}({{\mathbb {C}}})$.

6 This formula differs from the most general (0, 3)-geometry Dirac operator [3, Eq. 73] spanned by eight gamma matrices, since ours correspond to a simplification (also addressed in §V. A of op. cit.) byproduct of *V* being irreducible and the product of all gamma matrices being a scalar multiple of the identity.

7 The restrictions $0 < \sigma \le 3$ and $1<\nu \le 3$ account for the appearance in Eq. (3.2b) of exactly three gamma matrices whose indices are increasingly ordered, as in the characterization (2.7) of fuzzy Dirac operators. To match the canonical Dirac operator on a spinor bundle, one could redefine *H*, fully anti-symmetrize, and compare expressions.

8 Here we emphasize the composition to avoid potential confusion arising from the objects inside commutator already being (anti-)commutators themselves. Should no confusion arise, so we drop the $\circ$.

9 That group is isomorphic to ${\mathbb {R}}^+ \times \mathrm {U} (n_1) \times \mathrm {U} (n_2)$, but we will keep the full notation and the embedding for later convenience.

10 Here the fact that the unitary quaternions $\{ q\in \mathbb {H} : q^*q=1=qq^* \}$ are unimodular (i.e., their determinant is 1 in the embedding of $\mathbb {H}$ into $2\times 2$ matrices), $\mathcal {U} (\mathbb {H})\cong \mathrm {SU}(2)$, causes that unimodularity has influence on the $\mathbb {H}$ summand. That is why $\mu _3$ appears as fiber instead of $\mu _{3\times 2}$.

11 The next equation is based on van Suijlekom’s [57, §8.2.1].

12 The precise statement is that $\mathrm {ad}_{[{\mathsf {L}}_\mu ,{\mathsf {L}}_\nu ]}$ is the analogue of $[\partial _\mu , \partial _\nu ]$, but Jacobi identity used as in Eq. (5.7) allows one to state this in terms of $[{\mathsf {L}}_\mu ,{\mathsf {L}}_\nu ]$ only.

13 I thank the attentional, anonymous referee, who found in previous versions an inconsistent match between the fuzzy and smooth cases. This led to find the by far more closer correspondence Eq. (6.3), for which integration by parts (performed in a previous version) is not even needed.

14 To be strict, one has to swap also the anti-Hermiticity by the Hermiticity in the both lines of (8.3), but this is clearly fixed by an imaginary factor and is ignored here.

15 We recall that  is actually dependent on , since the operators  parametrize the inner fluctuations of the Dirac operator ${D_{\mathrm {f}}\otimes 1_F}$, itself parametrized by , but this dependence is not made explicit.

16 Tangentially, a discussion on gauge theories and the BV-formalism in the modern language of $L_\infty$-algebras appears in [16], in a noncommutative field theory (but also different) context.
