# Correction to: ‘On Multimatrix Models Motivated by Random Noncommutative Geometry II: A Yang-Mills-Higgs Matrix Model’

**DOI:** 10.1007/s00023-024-01456-9

**Published:** 2024-07-18

**Authors:** Carlos I. Perez-Sanchez

**Affiliations:** Institute for Theoretical Physics, University of Heidelberg, Philosophenweg 19, 69120 Heidelberg, Germany

**Correction to: Ann. Henri Poincaré** 10.1007/s00023-021-01138-w

Three equations in the body of Theorem 6.1 of [1] (present in the author’s submission) did not appear in the published article due to a mistake by the *Author Corrections Team* of *Springer Nature Journals Production*. In that theorem, the spectral action $\frac{1}{4}{{\,\textrm{Tr}\,}}_\mathcal {H}g(D)$ for polynomial *g* is computed in terms of several sectors. However, in the published version, only one of such, namely6.2appears defined. The correct group of equations (6.2), as the author submitted them, should have been displayed as: 6.2a6.2b6.2c6.2d (Secondarily: In the proof, the polynomial appearing in this errata in the spectral action $\frac{1}{4}{{\,\textrm{Tr}\,}}_\mathcal {H}g(D)$ was called *f* there; here, we use *g* in order not to give the impression that the ‘f’ in $S_{\textsc {ym}}^\textrm{f},\ldots ,S^\textrm{f}_\vartheta$ refers to the polynomial; straight ‘f’ rather means ‘fuzzy space’. The enormous flexibility of arXiv regarding the font choices takes care of this distinction; hence the polynomial in question is uniformly called *f* in the preprint version which is, and already before this errata was, correct.)
